# Culturable bacterial endophytes of *Aconitum carmichaelii* Debx. were diverse in phylogeny, plant growth promotion, and antifungal potential

**DOI:** 10.3389/fmicb.2023.1192932

**Published:** 2023-05-17

**Authors:** Lan Zou, Qian Wang, Muyi Li, Siyu Wang, Kunhao Ye, Wei Dai, Jing Huang

**Affiliations:** ^1^School of Life Science and Engineering, Southwest University of Science and Technology, Miangyang, China; ^2^Institute of Traditional Chinese Medicinal Materials, Miangyang Academy of Agricultural Science, Mianyang, China

**Keywords:** biological control, endophytic bacteria, genetic diversity, plant growth promotion, *Sclerotium rolfsii*

## Abstract

Medicinal plants harbor tremendously diverse bacterial endophytes that maintain plant growth and health. In the present study, a total of 124 culturable bacterial endophytes were isolated from healthy *Aconitum carmichaelii* Debx. plants. These strains were clustered into 10 genera based on full-length 16S rDNA sequences, among which *Bacillus* and *Pseudomonas* were the dominant genera. In addition, *A. carmichaelii* may capture 10 potential new bacterial species based on multi-locus sequence analysis of three housekeeping genes (*gyrA, rpoB*, and *atpD*). The majority of these bacterial endophytes exhibited plant growth-promoting ability through diverse actions including the production of either indole acetic acid and siderophore or hydrolytic enzymes (glucanase, cellulose, and protease) and solubilization of phosphate or potassium. A total of 20 strains inhibited hyphal growth of fungal pathogens *Sclerotium rolfsii* and *Fusarium oxysporum in vitro* on root slices of *A. carmichaelii* by the dual-culture method, among which *Pseudomonas* sp. SWUSTb-19 showed the best antagonistic activity. Field experiment confirmed that *Pseudomonas* sp. SWUSTb-19 significantly reduced the occurrence of southern blight and promoted plant biomass compared with non-inoculation treatment. The possible mode of actions for *Pseudomonas* sp. SWUSTb-19 to antagonize against *S. rolfsii* involved the production of glucanase, siderophore, lipopeptides, and antimicrobial volatile compounds. Altogether, this study revealed that *A. carmichaelii* harbored diverse plant growth-promoting bacterial endophytes, and *Pseudomonas* sp. SWUSTb-19 could be served as a potential biocontrol agent against southern blight.

## 1. Introduction

Plant bacterial endophytes are microorganisms that reside inside plant tissues for their part or whole life without causing apparent disease symptoms in the host (Wu et al., 2021). Bacterial endophytes can be found in different organs of almost all plants. Except for seed-borne endophytes, most endophytic bacteria were employed from soil or air by host plants (Eljounaidi et al., 2016). Plant endophytic bacterial composition exhibited host-specific properties and could be affected by environmental factors (Afzal et al., 2019; Xu et al., 2019; Wu et al., 2021). Nevertheless, *Bacillus* and *Pseudomonas* are dominant bacterial genera in many plants (Santoyo et al., 2016; Xu et al., 2019; Pang et al., 2022). Endophytic bacteria are considered to have a symbiotic relationship with plants (Sharma et al., 2020). In their interactions, plants provide nutrients and niches for bacteria, in return, bacterial endophytes play pivotal roles in promoting plant growth and tolerance against biotic and abiotic stresses (Gorai et al., 2021). Many types of research have focused on isolating and screening of functional bacterial endophytes either to promote plant growth or protect plants against pathogens. These types of bacterial endophytes are an important resource for biofertilizers and biocontrol agents. For example, *Bacillus* spp. strains isolated from mulberry showed great potential for plant growth promotion and antimicrobial activity (Xu et al., 2019). *P. aeruginosa* B18, a bacterial endophyte isolated from sugarcane, showed plant growth promoting and antifungal activities against sugarcane pathogens including *Sporisorium scitamineum, Ceratocystis paradoxa*, and *Fusarium verticillioides* (Singh et al., 2021). *Stenotrophomonas rhizophila* isolated from wheat enhanced plant growth and boosted plant defense against *F. pseudograminearum* (Liu et al., 2021). *Chitinophaga* and *Flavobacterium* were enriched by sugar beet to suppress the root pathogen *Rhizoctonia solani* (Carrion et al., 2019). The mechanisms for plant growth promotion by bacterial endophytes involve the acquisition of nutrients such as nitrogen, phosphate, potassium, and iron and the production of plant hormones such as auxin, cytokinin, or ethylene (Santoyo et al., 2016; Wu et al., 2021), while the production of hydrolytic enzymes and antimicrobial metabolites such as antibiosis and volatile organic compounds and induction of systemic resistance are mechanisms employed by bacterial endophytes for plant protection against pathogens (Abo-Elyousr et al., 2021; Wang et al., 2021; Dimkić et al., 2022).

*Aconitum carmichaelii* Debx. is a famous medicinal plant widely distributed and extensively used in Asian countries including China, Korea, Japan, and India (Miao et al., 2019). The most common part used as a gradient in many medical prescriptions is the processed lateral root of *A. carmichaelii*, called Fuzi (Chan et al., 2021). Fuzi is rich in diterpene alkaloids with anti-inflammation and analgesic action and anti-tumor and anti-aging activities (Zhou et al., 2015). Fuzi has been used to treat various human diseases such as rheumatoid arthritis, tumor, and depression, for over 2,000 years in traditional Chinese medicine (Wu et al., 2018). In China, *A. carmichaelii* is mainly planted in Sichuan, Yunnan, and Shaanxi provinces mainly as an economic crop. The term “Daodi” or geo-authentic area was used to characterize medicinal plants with the best quality. Jiangyou in Sichuan has been recognized as the geo-authentic area of *A. carmichaelii* for over 1,000 years (Yu et al., 2016). Special management during the growth period of *A. carmichaelii* were performed in the geo-authentic area, including the removal of redundant lateral roots (only two or three per plant were kept) and the top part of the plants. Except being widely used in China, Fuzi from Jiangyou has also been exported to Japan, India, and European countries, and the demand for Fuzi increase year by year (Fu et al., 2021).

Soil-borne diseases such as southern blight caused by *Sclerotium rolfsii* and root rot caused by *F. oxysporum* have severely hampered the sustainable development of *A. carmichelii*. Southern blight can cause up to 60% of yield loss of *A. carmichaelii* in Shaanxi area (Li et al., 2019b), and the situation was worse in geo-authentic areas where the wounds caused by removing the redundant lateral roots favored the infection by *S. rolfsii* (Dai et al., 2016). *S. rolfsii* usually infects the base of *A. carmichealii* stem causing wilt, rot, and then the death of the plant. In addition, *S. rolfsii* can form a special structure called sclerotium, which is generated from the aggregation of vegetative hyphae enclosed by a hard brown surface (Li et al., 2019b). These sclerotia can survive for a long time in harsh conditions and are resistant to fungicides (Li et al., 2019b), and they will germinate into hyphae once the situation was favorable. Therefore, it is difficult to control southern blight by either crop rotations or chemical fungicides (Li et al., 2020). In addition, in order to promote the yield of *A. carmichaelii*, large amount of chemical fertilizers was applied by farmers to the soil when cultivating *A. carmichaelii*. The overuse of chemical fertilizers facilitated the acidification of the soil which intensified the outbreak of soil-borne diseases (Dai et al., 2016).

Microbe-based biocontrol agents and biofertilizers have attracted great attention as an effective and environmentally friendly alternative for the control of soil-borne diseases (Javed et al., 2021; Khan and Javaid, 2022). For example, Li et al. (2019b, 2020) showed that one fungal strain (*Penicillium griseofulvum* CF3) and two actinobacterial strains (*Streptomyces pactum* Act12 and *S. rochei* D74) isolated from strawberry soil were able to reduce root disease occurrences of *A. carmichaelii* in Shaanxi area. However, to our best knowledge, systematic study on genetic diversity, plant growth promotion, and antifungal potential of bacterial endophytes isolated from *A. carmichaelii* has not been reported. To fill these knowledge gaps, the aims of the current study were to (1) isolate culturable bacterial endophytes from different tissues of *A. carmichaelii* collected from geo-authentic areas and uncover their genetic diversity; (2) assess plant growth promoting ability of these culturable endophytic bacteria *in vitro*; and (3) screen bacterial endophytes displaying antifungal activity against *S. rolfsii* and *F. oxysporum in vitro* and evaluate the corresponding biocontrol potential against southern blight of *A. carmichaelii* under field conditions. Ultimately, the goal of the current study was to maintain endophytic bacterial resources from *A. carmichaelii* and investigate their plant growth promoting and antimicrobial potential as biocontrol agents on *A. carmichaelii*.

## 2. Materials and methods

### 2.1. *A. carmichaelii* plants collection

Healthy *A. carmichaelii* plants were collected from Jiangyou, Sichuan province, where *A. carmichaelii* has been cultivated for over 1,000 years. A total of 10 sites were selected from the main planting areas of Jiangyou (Table 1), and five healthy plants without any disease symptoms were targeted in each site. A total of 150 plants were collected during the harvest time (June) of *A. carmichaelii* in 2020. Plants were scooped up from the soil gently without causing any physical wounds, stored in sterilized plastic bags, and transported to the laboratory within 24 h.

**Table 1:** Characterization of sampling sites for *A. carmichaelii* plants collection in Jiangyou area.

**Sampling sites**	**Longitude**	**Latitude**	**Elevation (m)**	**No. of isolates**	**Leaf**	**Root**	**Stem**
Yangting, Wudu, Jiangyou, and Sichuan	E 104° 47′ 31″	N 31° 53′ 20″	570	12	2	6	4
Laopingba, Sanhe, Jiangyou, and Sichuan	E 104° 47′ 04″	N 31° 49′ 44″	550	25	8	10	7
Baizhi, Sanhe, Jiangyou, and Sichuan	E 104° 45′ 40″	N 31° 49′ 17″	540	5	0	4	1
Changgeng, Zhangming, Jiangyou, and Sichuan	E 104° 42′ 43″	N 31° 42′ 32″	510	10	1	4	5
Changqing, Xiping, Jiangyou, and Sichuan	E 104° 37′ 10″	N 31° 42′ 29″	530	11	2	5	4
Yaoyue, Qinglian, Jiangyou, and Sichuan	E 104° 39′ 42″	N 31° 41′ 28″	520	8	3	3	2
Anzhou, Mianyang, and Sichuan	E 104° 27′ 71″	N 31° 55′ 17″	593	8	1	3	4
Qiaolou, Taiping, Jiangyou, and Sichuan	E 104° 41′ 44″	N 31° 42′ 34″	470	26	7	8	11
Puzhao, Taiping, Jiangyou, and Sichuan	E 104° 41′ 28″	N 31° 43′ 51″	469	8	0	7	1
Qingyi, Fucheng, Mianyang, and Sichuan	E 104° 41′ 43″	N 31° 32′ 1″	470	11	3	5	3
Total				124	27	55	42

### 2.2. Isolation of endophytic bacteria

Plants were washed throughout under tap water to remove soil and dust and dried with filter papers. Then, different parts (roots, stems, and leaves) were separated and cut into small slices or pieces for surface sterilization. In brief, materials were immersed in 75% alcohol for 2 min, followed by washing in sterilized water two times. NaClO (2%), then, was added for further sterilization (10 min), followed by washing with sterilized water five times. Aliquots (100 μL) from the last wash were spotted on Luria–Bertani (LB) agar medium and incubated at 28°C for 7 days to check the efficacy of surface sterilization. No colonies detected from the last wash indicated a successful surface sterilization, and the corresponding materials were used for the isolation of endophytic bacteria. Sterilized roots, stems, and leaves were crushed into smaller pieces by sterilized grinders, respectively. Then, the pieces were transferred to LB agar medium and incubated at 28°C for up to 14 days. Colonies around the pieces were picked and purified in new LB agar medium at least three times. Purified strains were stored in 25% glycerol at −80°C.

### 2.3. 16S rDNA-RFLP analysis

DNA was extracted from purified endophytic bacterial strains, following the procedure by Chen et al. (2018). Then, full length 16S rDNA was PCR amplified by the primer pairs 27F (5′-AGAGTTTGATCCTGGCTCAG-3′) and 1492R (5′-TACGGCTACCTTGTTACGAC TT-3′) in a 30 μL PCR reaction mixture by the following procedure: one cycle at 95°C for 5 min, followed by 30 cycles of 94°C for 30 s, 50°C for 30 s, 72°C for 2 min, and a final extension at 72°C for 10 min. The PCR products (~1,500 bp) were checked in 1% agarose gel and purified for restriction fragment length polymorphism (RFLP) analysis. Three restriction enzymes (*Hae*III, *Hinf* I, and *Taq*I) were used for the digestion of 16S rDNA, following the manufacturer's instructions (Fermentas, EU). Fragments from the digestion were separated by gel electrophoresis in 2% agarose at 80 V for 3 h and photographed. The 16S rDNA-RFLP analysis was carried out by combining the results of three restriction enzymes. Then, the cluster analysis of 16S rDNA-RFLP was conducted by the UPGM clustering algorithm in a NTSYS program (Chen et al., 2018).

### 2.4. Phylogenetic analysis of endophytic bacteria

According to 16S rDNA-RFLP, representative strains were selected for sequencing of housekeeping genes including 16S rRNA, *gyrA* (responsible for the synthesis of DNA gyrase subunit A), *rpoB* (encoding RNA polymerase subunit beta), and *atpD* (encoding ATP synthase beta subunit). 16S rRNA gene was amplified as mentioned before. *gyrA, rpoB*, and *atpD* were amplified as described in Supplementary Table 1. Then, PCR products were sent to Qingke Biotechnology Co., Ltd (Beijing, China) for sequencing. Sequences generated were blasted at National Center for Biotechnology Information (NCBI) database, and reference strains were selected. Phylogenetic analysis was conducted using the Neighbor-Joining method in Mega X with 1,000 bootstrapped replicates. Multilocus sequence analysis (MLSA) by concatenated sequences of *gyrA, rpoB*, and *atpD* was used to define genospecies of representative strains (Glaeser and Kampfer, 2015).

### 2.5. Plant growth-promoting ability analysis

Indole acetic acid (IAA) production, siderophore production, phosphate and potassium solubilization, and enzyme production (cellulase, protease, and glucanase) were assessed for plant growth-promoting ability of bacterial endophytes. A single colony of candidate strain was inoculated in liquid LB medium supplemented with 1 mL L-tryptophan (2.5 mg/mL) and then incubated at 28°C for 2 days. The supernatant (2 mL) was collected and added by 4 mL Salkowski reagent [1 mL FeCl_3_ (0.5 M) and 49 mL HClO_4_ (35% v:v)], well-vortexed, and incubated at room temperature under dark condition for 30 min. LB liquid medium (without bacteria) was used as a blank control following the same procedure. Then, the absorbance value at 530 nm was detected using a spectrophotometer (HZ170724-201). Commercial pure IAA (Sigma–Aldrich, United States) with different concentrations (0, 5, 10, 20, 40, and 60 mg/L) following the same procedure mentioned above was used to make a standard curve.

Cell suspension of each candidate strain was prepared by inoculating a single colony into LB liquid medium (5 mL) and incubating at 28°C at 150 rpm/min until OD_600_ = 1.0. Then, aliquot (10 μL, OD_600_ = 0.2) was dropped on chrome azurol-s (CAS) agar medium for siderophore production, Pikovskaya's agar medium containing Ca_3_(PO_4_)_2_ or Lecithin for phosphate solubilization, and Aleksandrow medium for potassium solubilization (Zhang and Kong, 2014; Xu et al., 2019). The production of protease, cellulase, and β-glucanase was carried out on skim milk agar medium, carboxymethyl cellulose (CMC) agar medium stained by congo red (0.2%, W:V), and glucan agar medium, respectively (Xu et al., 2019; Ben Khedher et al., 2021). The presence of a transparent zone or hydrolysis-induced halo around the bacterial colony indicated a positive activity of the strain after incubating at 28°C for 7 days.

### 2.6. Antifungal activity assessment

The dual-culture method was used to test the antifungal activity of endophytic bacteria both *in vitro* and on *A. carmichaelii* root slices. In this study, *S. rolfsii* and *F. oxysporum* strains were used as targeted pathogens (Li et al., 2020; Zou et al., 2023). Pathogens were inoculated on potato dextrose agar (PDA) medium and incubated at 25°C for 5 days. Then, one agar disc (2.0 mm diameter containing pathogenic hyphae) was transported to the center of a new PDA plate. Four droplets of cell suspension (10 μL) of candidate strain (described as mentioned above) were spotted on the PDA medium, 2.0 cm away from the disc in four directions. The PDA plate was incubated at 25°C for 7 days, and the diameter of the fungal pathogen was measured. Sterilized LB liquid medium was used as a negative control. All the tests were performed in triplicates. Strains that showed antagonistic activities *in vitro* were screened out for antifungal activity assessment on *A. carmichaelii* root slices. In brief, healthy *A. carmichaelii* roots without any physical wounds were washed, dried, and cut into 1.0 cm thick slices. These slices were surface sterilized as described before and placed onto a filter paper wetted with 1 ml of sterilized water in a sterilized Petri dish. Cell suspension (10 μL, OD_600_ = 0.2) of each candidate isolate was spotted on the center of the slice and dried at room temperature. Then, a pathogenic agar disc (2.0 mm diameter) was placed on the center of the slice. The Petri dish was, then, sealed by parafilm and incubated at 25°C for 7 days. Thereafter, the diameter of the pathogen was measured. Only pathogen disks co-cultured with sterilized LB broth medium (without bacteria) inoculated on the root slice were used as control. The experiments were carried out in triplicates. The inhibition rate was calculated by the following formula:


$$\begin{array}{l} \text{Inhibition rate }\left(\text{\%}\right)=\left(\text{Dc}-\text{Db}\right)/\text{Dc}\times \;100 \end{array}$$

where Dc was the diameter of the pathogen from the control trial, and Db was the diameter of the pathogen co-cultured with bacterial strain.

### 2.7. Effect of cell-free culture filtrate of SWUSTb-19 on hyphal growth, sclerotia formation, and germination of *S. rolfsii*

SWUSTb-19 was a stroke on LB agar medium and incubated at 28°C for 3 days. A single colony was picked, inoculated into 50 mL LB liquid medium, and incubated at 28°C at 150 rpm/min for 3 days to make cell culture. Then, the culture was filtrated by a 0.22 μM filtrating membrane to obtain cell-free culture filtrate. The filtrate was added to autoclaved warm PDA agar medium (1:4 v:v), mixed well, and poured into Petri dishes to make plates. *S. rolfsii* was inoculated on PDA plates and incubated at 28°C for 7 days to obtain mature sclerotia. Then, well-grown *S. rolfsii* disks or mature sclerotia (12 per plate) were transferred to the PDA plates containing cell-free culture of SWUSTb-19 and incubated at 28°C for up to 9 days. The diameter and number of sclerotia were recorded to evaluate the effect of cell-free culture of SWUSTb-19 on hyphal growth and sclerotia formation every 24 h. Number of germinated sclerotia was also recorded to evaluate the effect of cell-free culture of SWUSTb-19 on sclerotia germination every 24 h. PDA plates mixed with sterilized LB liquid medium were used as a control. The experiments were performed in triplicates. The inhibition rate was calculated as mentioned above.

### 2.8. Effect of volatile compounds produced by SWUSTb-19 on hyphal growth of *S. rolfsii*

Dual culture assay by sealing two agar base plates was carried out to test the effect of volatile compounds produced by SWUSTb-19 on the growth of *S. rolfsii*. One LB agar plate was inoculated with SWUSTb-19, and the other was PDA plate inoculated with a plug of *S. rolfsii*. The two plates were mouth-to-mouth sealed tightly by parafilm. Direct contact between the two strains was not accessible, except for the air in this equipment. Plates were incubated at 28°C for 7 days, and diameters of the pathogen were recorded every 24 h. An empty LB agar plate coupled with a PDA plate inoculated with *S. rolfsii* disc was used as control. The experiments were performed three times.

### 2.9. PCR amplification of lipopeptides and polyketides synthesize genes

The genomic DNA of SWUSTb-19 was extracted by a DNA isolation kit (B518225, Sango Biotech, Shanghai, China), following the manufacturer's instructions. Then, genes involved in the synthesis of fengycin (*fenA*), surfactin (*srfAA*), Bacilysin (*bac*), and bacillaene (*baeA*) were PCR amplified following the procedures and primers in the study by Ben Khedher et al. (2021). PCR products were checked by gel electrophoresis in 1% agarose.

### 2.10. Field trials

Field experiment was performed in the research field of Southwest University of Science and Technology (N 31° 32' 01”, E 104° 41' 43”, elevation 470 m a.s.l) in Jiangyou in 2021. Soil type was paddy soil in this field. The cultivar of *A. carmichaelii* used in the field experiment was MianFu No. 1, which was provided by the Mianyang academy of Agricultural Science. The field experiment followed the randomized block design principle. Each plot (1.0 × 3.7 m = 3.7 m^2^) contained two plant rows, and each row contained 25 plants. The row spacing was 30 cm, and the plant spacing was 14 cm. Four replicates were designed for each treatment. Fermentation culture was prepared as follows: a single colony of SWUSTb-19 was inoculated into 1 L LB liquid medium and incubated at 28°C with shaking (150 rpm/min) for 7 days until the concentration was over 1 × 10^9^ cell/ml. Then, the fermentation culture (10 ml/plant) was inoculated to the wounded parts of the root by a sterilized syringe after removing the redundant lateral roots of *A. carmichaelii* in late April. Roots were, then, covered with soil. Inoculation of sterilized LB liquid medium was used as a blank control. Other managements of *A. carmichelii* followed local customs. Southern blight disease occurrences were investigated consistently until harvest time in June. Plants displaying wilt, rot, or death accompanied by white hyphae or brown sclerotia were defined as southern blight symptoms, and the number of which were recorded. After harvesting, five healthy plants per plot were randomly collected to evaluate plant growth-promoting ability of SWUSTb-19 by measuring the fresh and dry weights of stem, main root, and lateral roots. The disease occurrence and control efficacy were calculated by the following formulas:


$$\begin{array}{l} \text{Disease occurrence }\left(\text{\%}\right)=\text{Nd}/\text{Nt}\times \;100 \end{array}$$

where Nd indicated the number of diseased plants, and Nt was the number of total plants.


$$\begin{array}{l} \text{Control efficacy }\left(\text{\%}\right)=\left(\text{DOc}-\text{DOt}\right)/\text{DOc}\times \;100 \end{array}$$

where DOc was the disease occurrence in CK, and DOt was the disease occurrence in SWUSTb-19 treatment.

### 2.11. Statistical analysis

Data of control efficacy by volatile compounds *in vitro*, effects of SWUSTb-19 cell culture on southern blight disease, and plant growth promotion under field conditions were analyzed by one-way analysis of variance (ANOVA), with least significant difference (LSD) test or Student's *t*-test. The difference was considered significant when *p* < 0.05. All statistical analyses were performed in SPSS V 25.0 (SPSS Inc. Chicago, United States). Housekeeping gene sequences (16S rRNA, *atpD, gyrA*, and *rpoB*) of representative strains were all deposited in the GenBank database under the accession numbers OP218645-OP218666, OP236689-OP236694, OP341255-OP341259, and OP296411-OP296500.

## 3. Results

### 3.1. Isolation of endophytic bacterial strains

A total of 124 endophytic bacterial strains were isolated from different organs of *A. carmichaelii* plants collected from 10 sites in the geo-authentic area. In total, 55 strains were isolated from roots, 42 strains were isolated from stems, and only 27 strains were obtained from leaves (Table 1).

### 3.2. Genetic diversity and phylogeny of endophytic bacteria

**Figure 1:**
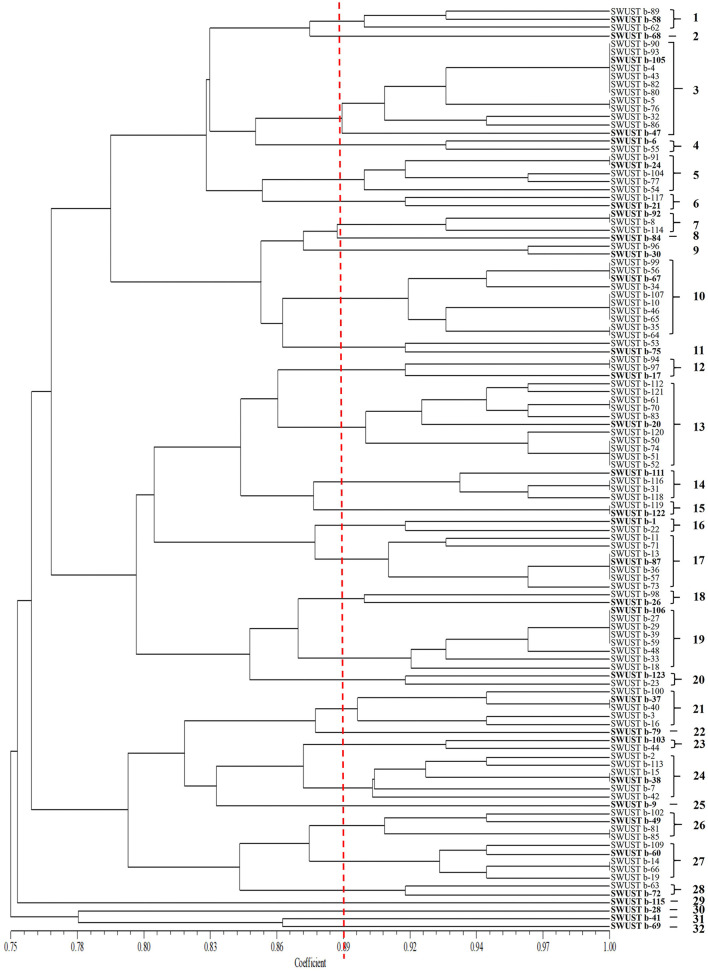
Cluster analysis of 16S rDNA-RFLP by three restriction enzymes (*Taq*I, *Hinf*I, and *Hae*III) of endophytic bacterial strains isolated from *A. carmichaelii*. The RFLP groups were labeled with Arabic numbers on the right.

**Figure 2:**
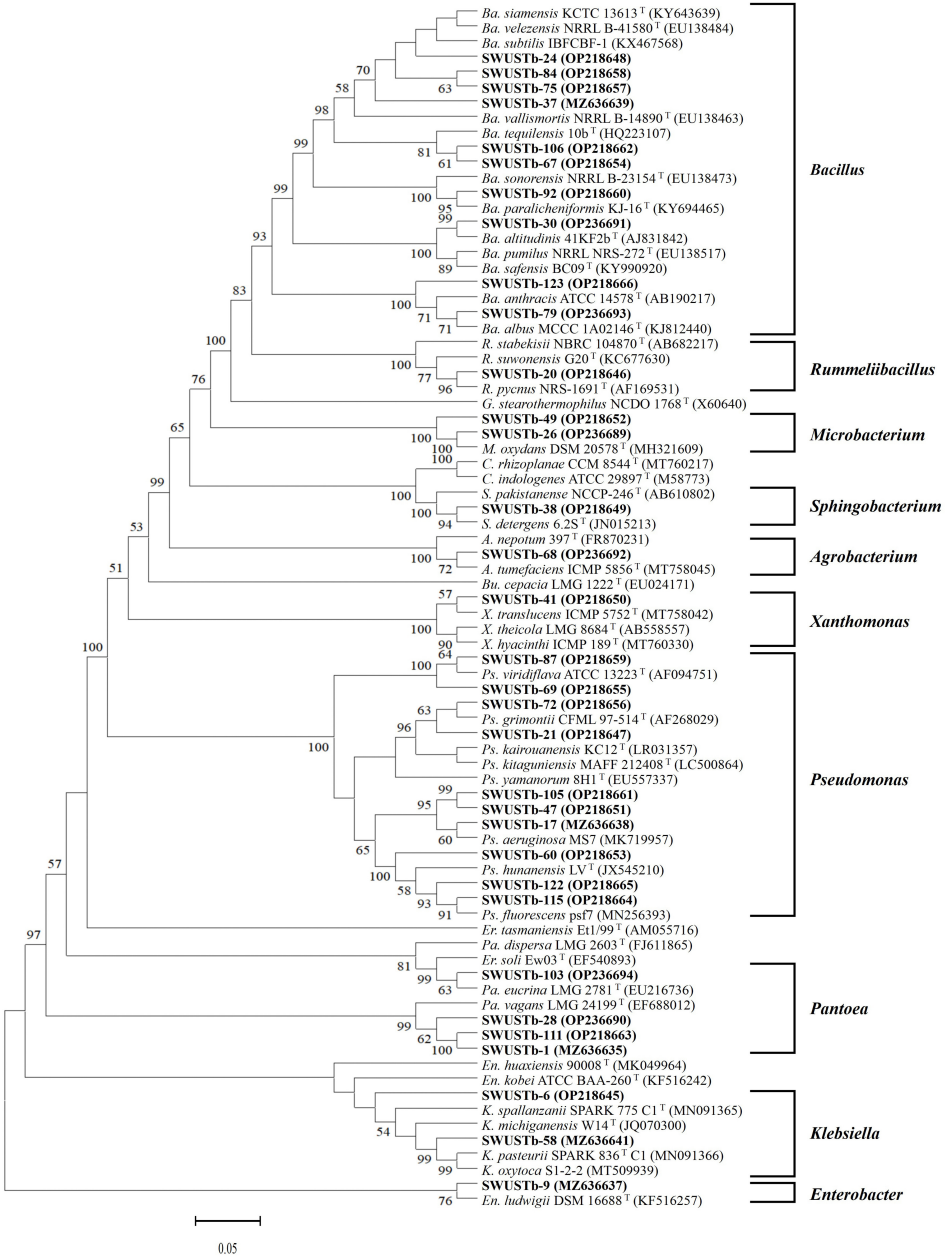
Phylogenetic tree based on 16S rRNA gene (1,400 bp) of endophytic bacterial strains and reference strains by the Neighbor-Joining method. Scar bar = 5% substitutions per site. Bootstrap values of >50% were indicated on the branches. *Ps, Pseudomonas; Pa, Pantoea; Er, Erwinia; En, Enterobacter; K, Klebsiella; Bu, Burkholderia; X, Xanthomonas; A, Agrobacterium; M, Microbacterium; G, Geobacillus; R, Rummeliibacillus; Ba, Bacillus; S, Sphingobacterium; C, Chryseobacterium*. GenBank accession numbers are in the brackets.

**Figure 3:**
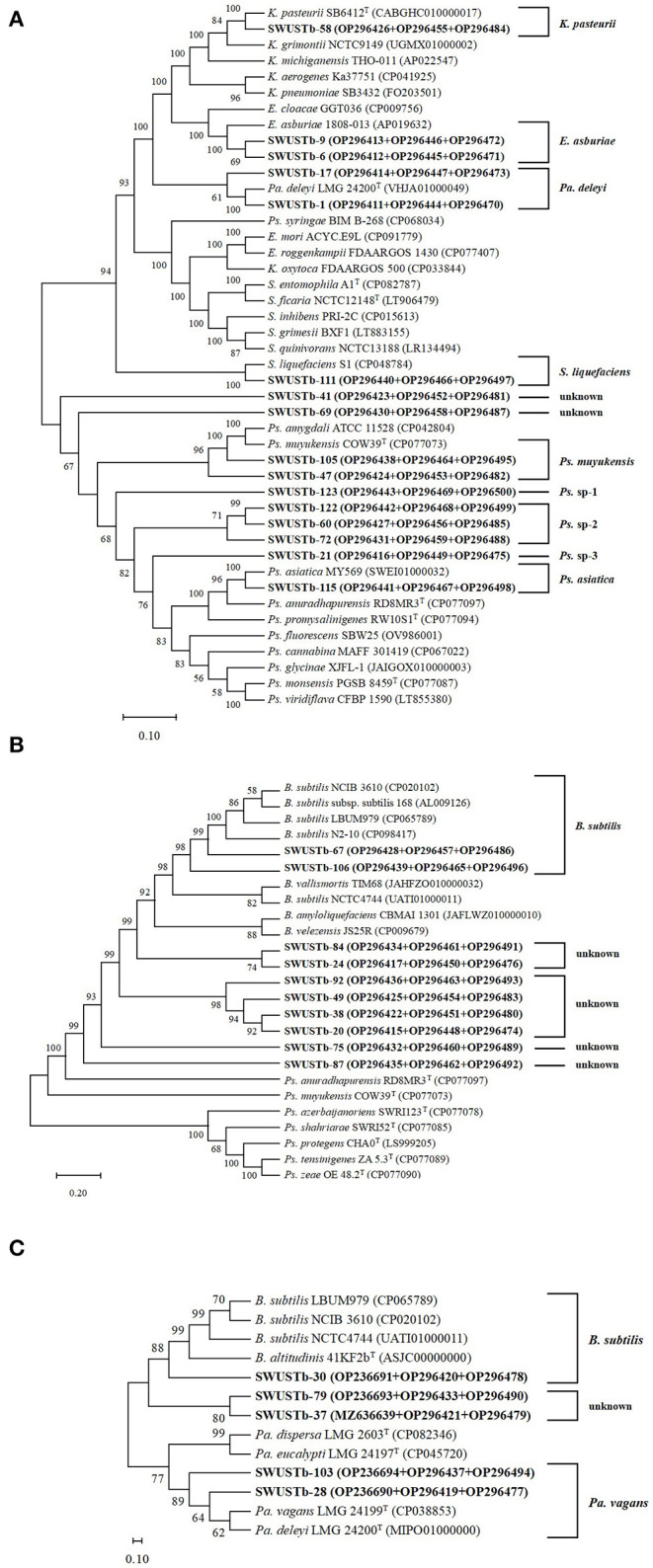
Neighbor-Joining trees based on multilocus sequence analysis using the concatenated sequences of **(A)** *atpD* (600 bp), *gyrA* (700 bp), and *rpoB* (1,000 bp) genes, **(B)** *atpD* (600 bp), *gyrA* (700 bp), and *rpoB* (500 bp) genes, and **(C)** 16S rRNA (1,400 bp), *atpD* (600 bp), and *ropB* (1,000 bp) genes of representative strains isolated from *A. carmichaelii* (in bold) and reference strains. Genbank accession numbers were in parentheses. Bootstrap values of >50% were shown on the branches. *K, Klebsiella; E, Enterobacter; Pa, Pantoea; Ps, Pseudomonas; S, Serratia; B, Bacillus*.

Amplification of 16S rDNA from bacterial genomic DNA resulted in a 1,500 bp fragment. A total of 114 strains succeeded in amplifying 16S rDNA and were used as materials for RFLP analysis. In general, a total of 13, seven, and nine types of digestion patterns were obtained by *Hinf* I, *Hea*III, and *Taq*I, respectively (Supplementary Table 2). By combining the digesting results of all three restriction enzymes, 114 strains were clustered into 32 groups at 89% similarity level (Figure 1). A total of 33 representative strains were selected for sequencing of housekeeping genes. According to Neighbor-Joining tree based on full length 16S rRNA gene sequence, endophytic bacteria of *A. carmichaelii* were clustered into 10 genera belonging to *Pseudomonas, Pantoea, Enterobacter, Klebsiella, Xanthomonas, Agrobacterium, Microbacterium, Rummeliibacillus, Bacillus*, and *Sphingobacterium* (Figure 2). *Bacillus* and *Pseudomonas* were the dominant genera, accounting for 34.2 and 30.7% of total strains, respectively. A total of 25 representative strains were able to amplify three housekeeping genes, five strains were able to amplify two genes (*atpD* and *rpoB*), and two strains could only amplify one housekeeping gene (*atpD*). Therefore, MLSA trees were constructed based on concatenated sequences of either *atpD, gyrA*, and *rpoB* (25 representative strains) or 16S rRNA, *atpD*, and *rpoB* (five strains). It is noteworthy that we used two pairs of primers to amplify the *rpoB* gene (Supplementary Table 1). Primer pair rpoBf/rpoBr generated a length of 1,000 bp PCR products while rpoB CM-1/CM31 generated a length of 500 bp PCR products, and no overlap was found between the two products. Therefore, MLSA trees were also constructed separately based on the primer pairs used for the *rpoB* gene. Collectively, three MLSA trees were constructed (Figure 3). The results by MLSA were preliminary and consistent with that of the 16S rRNA gene. For instance, SWUSTb-58 clustered closely with *K. pasteurii* SB6412^T^, and this strain could be assigned as *K. pasteurii*. SWUSTb-9 and SWUSTb-6 clustered with *E. asburia* 1808-013. SWUSTb-1 clustered with *Pantoea deleyi* LMG 24200^T^ (Figure 3A). However, a total of 17 representative strains clustered separately into 10 groups (Figure 3). For example, SWUSTb-41 and SWUSTb-69 clustered alone; therefore, we did not know their exact taxonomic status (Figure 3A). SWUSTb-123, SWUSTb-122, SWUSTb-60, SWUSTb-72, and SWUSTb-21 clustered separately into the *Pseudomonas* genus, so we assigned them as *Pseudomonas* spp. (Figure 3A). While SWUSTb-84, SWUSTb-24, SWUSTb-92, SWUSTb-49, SWUSTb-38, SWUSTb-20, SWUSTb-75, and SWUSTb-87 clustered separately with both *Bacillus* and *Pseudomonas*, their taxonomic status remained unknown (Figure 3B). SWUSTb-79 and SWUSTb-37 cluster alone as well (Figure 3C). In this situation, the taxonomic status of these strains was defined at the genus level, as presented in Supplementary Table 2.

### 3.3. Plant growth promoting characterization of bacterial endophytes isolated from *A. carmichaelii*

All 124 strains were tested for their plant growth-promoting potential *in vitro*. A total of 99 isolates were able to produce IAA, among which 25 strains produced relatively higher amount of IAA (>20 mg/L), and strain *Sphingobacterium* sp. SWUSTb-7 produced the highest amount of IAA (65.79 mg/L; Table 2). In total, 50 isolates showed the capacity to produce siderophore. There were 20, two, and four strains showing the capacity of solubilizing organic phosphate, inorganic phosphate, and potassium, respectively. In addition, 51, 34, and 62 strains were able to produce glucanase, cellulase, and protease, respectively (Table 2).

**Table 2:** Characterization of plant growth promotion property and enzyme production by culturable endophytic bacteria isolated from *A. carmichaelii*.

**Isolates**	**IAA (mg/L)**	**Siderophore**	**OP-solubilization**	**IP-solubilization**	**K-solubilization**	**Glucanase**	**Cellulose**	**Protease**
SWUSTb-1	21.88 ± 0.54	-	2.20 ± 0.00	-	-	1.76 ± 0.05	-	-
SWUSTb-2	41.55 ± 0.11	-	2.00 ± 0.51	-	-	-	-	-
SWUSTb-3	29.82 ± 2.39	-	-	-	-	-	-	-
SWUSTb-4	-	-	-	-	-	1.39 ± 0.05	-	2.20 ± 0.04
SWUSTb-5	26.66 ± 0.54	-	-	-	-	-	-	-
SWUSTb-6	41.34 ± 2.50	-	-	-	-	1.27 ± 0.01	-	-
SWUSTb-7	65.79 ± 0.76	-	-	-	-	1.42 ± 0.04	-	-
SWUSTb-8	59.65 ± 5.16	-	-	-	-	1.49 ± 0.09	-	-
SWUSTb-9	28.95 ± 0.54	-	-	-	-	1.34 ± 0.06	-	-
SWUSTb-10	3.84 ± 1.74	-	-	-	-	3.52 ± 0.03	3.41 ± 0.04	4.38 ± 0.13
SWUSTb-11	13.02 ± 0.27	-	-	-	-	-	-	-
SWUSTb-12	7.91 ± 0.71	-	-	-	-	-	-	-
SWUSTb-13	12.91 ± 0.60	3.05 ± 0.13	-	-	-	-	-	3.39 ± 0.35
SWUSTb-14	2.10 ± 0.33	1.05 ± 0.01	-	-	-	2.10 ± 0.16	-	4.10 ± 0.10
SWUSTb-15	18.08 ± 1.20	-	1.69 ± 0.17	-	-	-	-	-
SWUSTb-16	16.83 ± 0.92	-	-	-	-	-	-	-
SWUSTb-17	24.05 ± 0.54	-	-	-	-	-	1.17 ± 0.35	2.33 ± 0.13
SWUSTb-18	9.23 ± 0.48	-	-	-	-	1.30 ± 0.02	-	2.72 ± 0.12
SWUSTb-19	-	2.58 ± 0.02	-	-	-	1.61 ± 0.04	-	-
SWUSTb-20	4.65 ± 0.82	3.00 ± 0.17	-	-	-	-	-	1.82 ± 0.04
SWUSTb-21	2.37 ± 0.16	1.21 ± 0.00	-	-	-	-	-	3.68 ± 0.04
SWUSTb-22	3.62 ± 0.76	2.71 ± 0.00	-	-	2.92 ± 0.08	-	-	2.48 ± 0.05
SWUSTb-23	1.07 ± 0.38	-	-	-	3.86 ± 0.31	-	-	-
SWUSTb-24	2.86 ± 0.33	2.00 ± 0.00	-	-	-	3.58 ± 0.16	3.63 ± 0.07	3.36 ± 0.11
SWUSTb-25	13.29 ± 1.20	-	-	-	-	-	-	4.24 ± 0.33
SWUSTb-26	4.05 ± 1.20	3.13 ± 0.25	-	-	-	-	2.37 ± 0.13	-
SWUSTb-27	0.96 ± 0.16	2.17 ± 0.00	-	-	-	1.31 ± 0.03	-	2.41 ± 0.03
SWUSTb-28	17.60 ± 0.26	-	2.00 ± 0.00	-	-	1.74 ± 0.15	-	-
SWUSTb-29	5.09 ± 1.14	-	-	-	-	1.54 ± 0.07	1.72 ± 0.11	1.66 ± 0.07
SWUSTb-30	2.91 ± 0.38	2.86 ± 0.23	-	-	-	1.30 ± 0.02	-	1.98 ± 0.25
SWUSTb-31	-	-	-	-	-	2.44 ± 0.20	-	-
SWUSTb-32	0.47 ± 0.22	1.83 ± 0.33	-	-	-	-	3.86 ± 0.18	-
SWUSTb-33	11.17 ± 0.38	3.45 ± 0.06	-	-	-	3.00 ± 0.12	-	3.48 ± 0.10
SWUSTb-34	-	-	2.07 ± 0.30	-	-	1.28 ± 0.04	-	-
SWUSTb-35	-	1.17 ± 0.08	-	-	-	-	2.50 ± 0.29	1.23 ± 0.02
SWUSTb-36	-	2.32 ± 0.43	-	-	-	-	-	-
SWUSTb-37	22.21 ± 0.87	-	1.67 ± 0.19	-	-	1.50 ± 0.16	1.43 ± 0.03	-
SWUSTb-38	1.72 ± 0.05	2.60 ± 0.15	-	-	-	2.28 ± 0.33	-	3.76 ± 0.06
SWUSTb-39	4.22 ± 0.60	-	-	-	-	1.39 ± 0.06	2.18 ± 0.50	2.08 ± 0.12
SWUSTb-40	5.52 ± 0.60	2.29 ± 0.00	-	-	-	1.44 ± 0.09	1.37 ± 0.05	2.08 ± 0.10
SWUSTb-41	0.29 ± 0.29	4.27 ± 0.00	-	-	-	5.41 ± 0.15	-	4.88 ± 0.71
SWUSTb-42	7.60 ± 0.59	-	-	-	-	1.72 ± 0.07	-	3.65 ± 0.32
SWUSTb-43	3.29 ± 0.00	3.71 ± 0.04	-	-	-	1.40 ± 0.03	2.23 ± 0.01	1.78 ± 0.02
SWUSTb-44	34.65 ± 1.47	2.67 ± 0.00	1.96 ± 0.12	-	-	-	-	-
SWUSTb-45	-	-	-	-	-	1.49 ± 0.04	1.44 ± 0.04	2.00 ± 0.05
SWUSTb-46	2.42 ± 0.11	-	-	-	-	3.15 ± 0.00	3.55 ± 0.17	2.57 ± 0.05
SWUSTb-47	-	-	-	-	-	-	-	3.16 ± 0.12
SWUSTb-48	4.27 ± 0.22	3.05 ± 0.03	-	-	-	1.22 ± 0.02	-	1.33 ± 0.06
SWUSTb-49	32.53 ± 0.33	3.45 ± 0.14	-	-	-	-	-	-
SWUSTb-50	-	-	-	-	-	-	-	-
SWUSTb-51	-	-	-	-	-	-	-	-
SWUSTb-52	-	-	-	-	-	-	-	-
SWUSTb-53	24.73 ± 0.96	2.71 ± 0.02	-	-	-	1.88 ± 0.13	2.01 ± 0.80	1.87 ± 0.10
SWUSTb-54	-	3.63 ± 0.00	-	-	-	-	-	-
SWUSTb-55	8.24 ± 0.38	-	3.33 ± 0.22	-	-	-	-	-
SWUSTb-56	-	3.57 ± 0.22	-	-	-	1.50 ± 0.05	3.10 ± 0.21	4.50 ± 0.44
SWUSTb-57	13.40 ± 0.76	-	-	-	-	-	-	-
SWUSTb-58	45.63 ± 0.05	1.29 ± 0.01	-	-	-	-	-	-
SWUSTb-59	3.35 ± 0.05	3.66 ± 0.29	-	-	-	1.52 ± 0.04	1.58 ± 0.06	1.90 ± 0.08
SWUSTb-60	21.39 ± 0.16	-	-	-	-	-	-	-
SWUSTb-61	-	3.93 ± 0.24	-	-	-	1.30 ± 0.02	-	-
SWUSTb-62	42.53 ± 0.87	-	-	-	-	-	-	-
SWUSTb-63	4.16 ± 0.65	-	-	-	-	1.89 ± 0.11	-	3.87 ± 0.23
SWUSTb-64	-	-	-	-	-	1.64 ± 0.12	1.96 ± 0.15	-
SWUSTb-65	-	3.46 ± 0.08	-	-	-	-	-	3.07 ± 0.35
SWUSTb-66	18.89 ± 5.71	-	-	-	-	-	-	-
SWUSTb-67	3.40 ± 0.22	3.50 ± 0.00	-	-	-	1.67 ± 0.02	3.81 ± 0.04	2.73 ± 0.05
SWUSTb-68	14.76 ± 0.38	-	-	-	-	1.56 ± 0.04	-	-
SWUSTb-69	5.30 ± 0.49	-	-	-	-	-	-	2.68 ± 0.26
SWUSTb-70	13.78 ± 2.23	-	3.02 ± 0.23	-	-	-	-	-
SWUSTb-71	-	2.71 ± 0.00	-	-	-	-	-	2.22 ± 0.19
SWUSTb-72	7.75 ± 1.41	-	-	-	-	-	-	3.84 ± 0.15
SWUSTb-73	3.24 ± 0.05	2.48 ± 0.02	-	-	-	-	-	-
SWUSTb-74	2.80 ± 0.05	-	-	-	-	-	-	2.91 ± 0.07
SWUSTb-75	3.67 ± 0.71	3.19 ± 0.20	-	-	-	1.64 ± 0.23	2.52 ± 0.19	2.46 ± 0.07
SWUSTb-76	-	-	-	-	-	-	-	-
SWUSTb-77	4.60 ± 0.11	2.96 ± 0.11	-	-	-	-	-	4.14 ± 0.33
SWUSTb-78	14.27 ± 0.33	-	-	-	1.75 ± 0.00	1.32 ± 0.04	-	2.60 ± 0.10
SWUSTb-79	17.53 ± 0.33	2.04 ± 0.04	1.83 ± 0.17	-	-	-	1.85 ± 0.08	-
SWUSTb-80	-	-	-	-	-	-	-	1.18 ± 0.01
SWUSTb-81	-	3.45 ± 0.05	-	-	-	-	-	2.24 ± 0.10
SWUSTb-82	7.21 ± 0.76	-	-	-	2.00 ± 0.15	-	7.56 ± 1.26	2.41 ± 0.03
SWUSTb-83	3.18 ± 0.11	-	2.72 ± 0.15	-	-	-	-	-
SWUSTb-84	4.49 ± 2.28	-	-	-	-	3.63 ± 0.14	4.45 ± 0.09	3.89 ± 0.32
SWUSTb-85	22.53 ± 2.07	2.15 ± 0.16	1.71 ± 0.15	-	-	1.79 ± 0.11	-	3.52 ± 0.05
SWUSTb-86	28.29 ± 4.57	2.59 ± 0.00	-	-	-	1.75 ± 0.13	-	-
SWUSTb-87	6.61 ± 0.49	4.30 ± 0.10	-	-	-	-	-	3.58 ± 0.10
SWUSTb-88	0.14 ± 0.54	-	-	-	-	-	-	2.64 ± 0.17
SWUSTb-89	53.84 ± 0.54	-	4.89 ± 0.11	1.53 ± 0.07	-	-	-	-
SWUSTb-90	8.84 ± 0.54	-	-	-	-	1.49 ± 0.09	1.59 ± 0.11	2.82 ± 0.06
SWUSTb-91	11.23 ± 1.96	-	2.44 ± 0.23	-	-	1.49 ± 0.08	-	-
SWUSTb-92	-	1.23 ± 0.00	-	-	-	2.47 ± 0.16	3.13 ± 0.07	2.23 ± 0.20
SWUSTb-93	1.50 ± 0.05	3.82 ± 0.00	-	-	-	2.51 ± 0.33	-	2.25 ± 0.11
SWUSTb-94	-	-	-	-	-	1.52 ± 0.10	3.44 ± 0.69	-
SWUSTb-95	-	-	-	-	-	1.32 ± 0.04	1.52 ± 0.04	1.69 ± 0.09
SWUSTb-96	4.43 ± 0.49	3.76 ± 0.23	-	-	-	-	2.79 ± 0.50	3.11 ± 0.46
SWUSTb-97	35.50 ± 0.95	-	-	-	-	-	-	-
SWUSTb-98	36.83 ± 1.36	-	-	-	-	3.73 ± 0.96	-	-
SWUSTb-99	0.85 ± 0.16	-	-	-	-	2.60 ± 0.03	-	2.19 ± 0.30
SWUSTb-100	24.05 ± 0.43	-	2.19 ± 0.44	-	-	-	-	-
SWUSTb-101	6.07 ± 0.82	-	-	-	-	2.26 ± 0.13	3.00 ± 0.00	3.67 ± 0.35
SWUSTb-102	40.90 ± 1.09	-	-	-	-	-	-	-
SWUSTb-103	40.74 ± 2.01	3.00 ± 0.00	3.53 ± 0.30	1.00 ± 0.51	-	1.54 ± 0.14	2.18 ± 0.32	-
SWUSTb-104	14.93 ± 0.75	-	-	-	-	-	-	2.96 ± 0.47
SWUSTb-105	84.43 ± 7.88	-	-	-	-	-	-	-
SWUSTb-106	11.61 ± 0.16	1.17 ± 0.08	-	-	-	-	4.11 ± 0.32	2.36 ± 0.20
SWUSTb-107	2.86 ± 1.09	2.89 ± 0.19	-	-	-	-	1.68 ± 0.14	2.65 ± 0.17
SWUSTb-108	8.95 ± 0.11	-	-	-	-	-	-	-
SWUSTb-109	-	-	-	-	-	-	-	-
SWUSTb-110	0.20 ± 0.05	2.20 ± 0.00	-	-	-	-	-	-
SWUSTb-111	12.15 ± 2.55	-	-	-	-	1.47 ± 0.07	-	2.07 ± 0.21
SWUSTb-112	1.66 ± 0.11	-	-	-	-	-	-	1.84 ± 0.15
SWUSTb-113	13.67 ± 3.64	-	-	-	-	-	-	-
SWUSTb-114	0.04 ± 0.00	2.24 ± 0.34	-	-	-	1.39 ± 0.05	2.66 ± 0.21	1.75 ± 0.16
SWUSTb-115	5.79 ± 0.65	-	-	-	-	-	-	-
SWUSTb-116	6.93 ± 0.16	-	2.67 ± 0.08	-	-	-	-	3.71 ± 0.49
SWUSTb-117	6.23 ± 0.43	-	-	-	-	1.45 ± 0.04	3.29 ± 0.37	2.36 ± 0.30
SWUSTb-118	11.61 ± 0.71	-	-	-	-	-	-	-
SWUSTb-119	9.98 ± 0.16	1.76 ± 0.00	1.92 ± 0.08	-	-	-	-	-
SWUSTb-120	2.86 ± 0.33	3.68 ± 0.28	-	-	-	1.38 ± 0.03	4.02 ± 0.22	2.07 ± 0.16
SWUSTb-121	12.91 ± 2.01	1.44 ± 0.00	2.89 ± 0.41	-	-	-	-	-
SWUSTb-122	7.75 ± 0.43	-	2.56 ± 0.23	-	-	-	-	-
SWUSTb-123	-	3.18 ± 0.08	-	-	-	2.11 ± 0.06	-	-
SWUSTb-124	2.70 ± 0.16	-	-	-	-	-	-	-

Siderophore production, organic phosphate solubilization, inorganic phosphate solubilization, potassium solubilization, glucan degrading enzyme, cellulase and protease production abilities of corresponding strains were indicated by the ratio of siderophore diameter (cm), lecithin solubilization diameter (cm), Ca_3_(PO_4_)_2_ solubilization diameter (cm), K_2_O·Al_2_O_3_·6SiO_2_ solubilization diameter (cm), glucan degrading diameter (cm), cellulase diameter (cm), and protease diameter (cm) and bacterial colony diameter (cm), respectively—means not detected.

### 3.4. Antagonistic activity of endophytic bacterial isolates against pathogens

**Figure 4:**
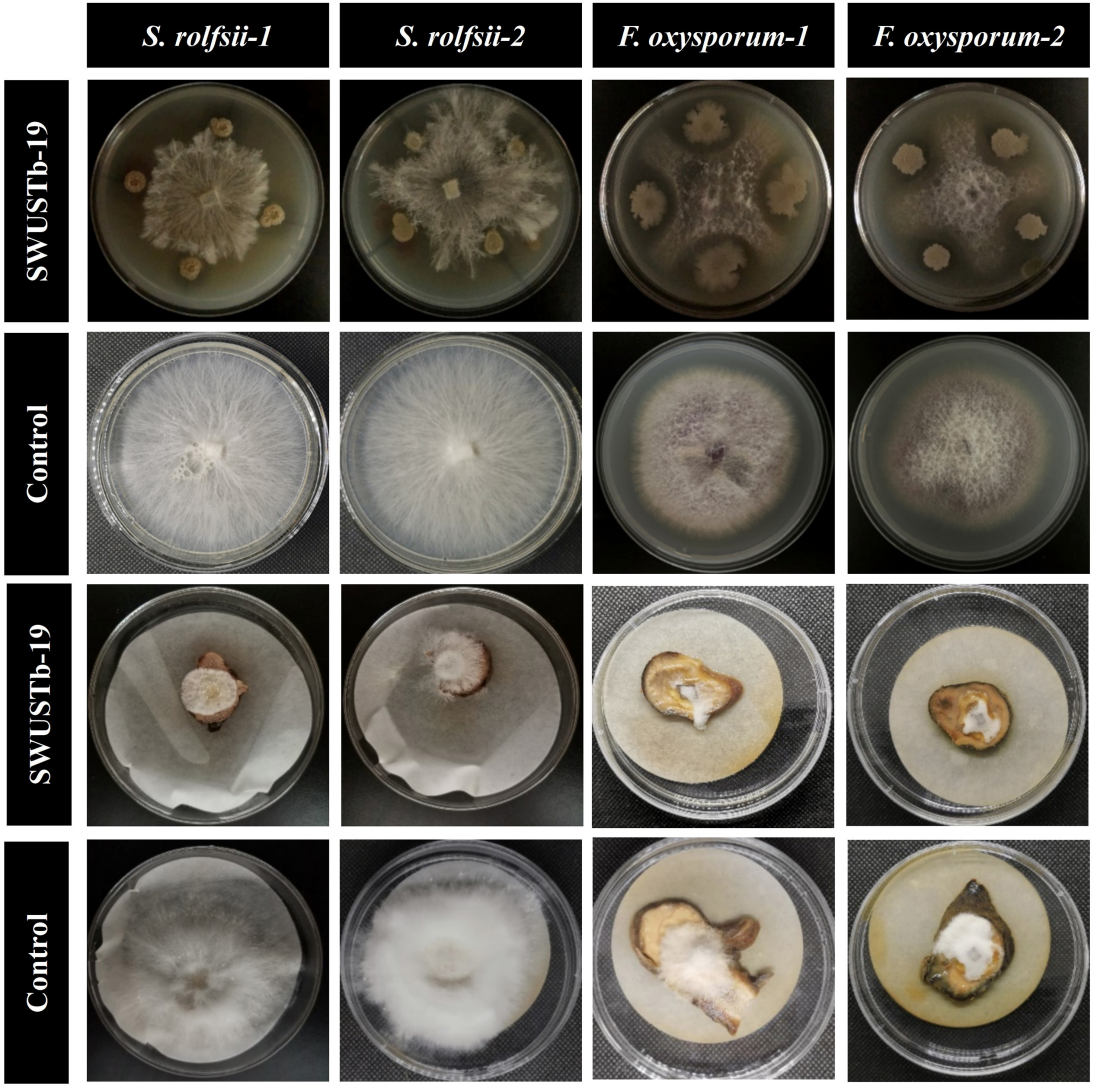
Antagonistic activity of SWUSTb-19 against *S. rolfsii* and *F. oxysporum in vitro* and on root slices of *A. carmichaelii*.

In total, 10 out of 124 strains showed significant antagonistic activities against the hyphal growth of *S. rolfsii*, which causes southern blight both *in vitro* and on root slices of *A. carmichaelii*. A total of 20 strains showed strong antagonistic activity against *F. oxysporum*, which causes root rot *in vitro*, among which 16 strains also displayed strong inhibiting effects on the hyphal growth of *F. oxysporum* and on root slices of *A. carmichaelii*. *Pseudomonas* sp. SWUSTb-19 exhibited the strongest inhibiting ability against both *S. rolfsii* and *F. oxysporum in vitro* and on root slices of *A. carmichaelii*, among all the strains (Table 3 and Figure 4) and therefore was used as material for the following experiments.

**Table 3:** Antagonistic activities of endophytic bacteria against root rot and southern blight pathogens of *A. carmichaelii*.

**Isolate**	**Inhibition rate on PDA plate (%)**				**Inhibition rate on** ***A. carmichaelii*** **root slice (%)**			
	***S. rolfsii*** **-1**	***S. rolfsii*** **-2**	***F. oxysporum*** **-1**	***F. oxysporum*** **-2**	***S. rolfsii*** **-1**	***S. rolfsii*** **-2**	***F. oxysporum*** **-1**	***F. oxysporum*** **-2**
SWUST b-10	61.86 ± 0.03	-	53.60 ± 0.03	53.64 ± 0.02	17.02 ± 0.54	-	18.56 ± 0.09	27.59 ± 0.15
SWUST b-19	63.75 ± 0.62	60.54 ± 0.03	55.13 ± 0.03	52.90 ± 0.03	30.00 ± 0.58	53.25 ± 0.07	54.17 ± 0.09	30.34 ± 0.06
SWUST b-20	-	-	45.94 ± 0.03	-	-	-	-	-
SWUST b-23	-	-	40.73 ± 0.03	39.38 ± 0.03	-	-	-	-
SWUST b-24	53.13 ± 0.12	51.30 ± 0.02	52.07 ± 0.03	44.58 ± 0.03	16.32 ± 0.92	49.68 ± 0.35	42.19 ± 0.03	-
SWUST b-32	46.87 ± 0.03	-	39.20 ± 0.09	48.44 ± 0.03	17.89 ± 0.32	-	18.56 ± 0.06	29.56 ± 0.15
SWUST b-38	-	-	-	58.84 ± 0.03	-	-	-	-
SWUST b-44	-	-	-	38.04 ± 0.03	-	-	-	-
SWUST b-46	51.48 ± 0.06	55.33 ± 0.03	-	62.41 ± 0.03	25.79 ± 0.09	55.97 ± 0.69	-	20.20 ± 0.06
SWUST b-53	49.59 ± 0.07	-	51.45 ± 0.03	53.49 ± 0.06	18.07 ± 0.09	-	21.10 ± 0.03	-
SWUST b-56	-	-	43.80 ± 0.03	52.90 ± 0.03	-	-	10.13 ± 0.18	9.85 ± 0.07
SWUST b-64	51.95 ± 0.09	-	45.94 ± 0.03	46.95 ± 0.03	32.10 ± 0.09	-	60.34 ± 0.03	-
SWUST b-75	-	-	49.31 ± 0.06	62.41 ± 0.03	-	-	14.35 ± 0.09	-
SWUST b-84	46.04 ± 0.03	40.40 ± 0.03	42.88 ± 0.03	53.49 ± 0.03	23.33 ± 0.97	62.26 ± 0.26	5.91 ± 0.03	12.81 ± 0.03
SWUST b-92	-	-	42.88 ± 0.03	32.69 ± 0.03	-	-	42.19 ± 0.15	5.91 ± 0.12
SWUST b-99	-	-	46.86 ± 0.03	49.03 ± 0.03	-	-	39.66 ± 0.12	4.93 ± 0.07
SWUST b-101	62.10 ± 0.06	59.36 ± 0.03	47.47 ± 0.03	55.87 ± 0.06	26.14 ± 0.65	28.72 ± 0.75	12.66 ± 0.13	22.66 ± 0.12
SWUST b-106	62.57 ± 0.03	-	60.64 ± 0.03	63.74 ± 0.06	32.80 ± 0.32	-	35.44 ± 0.09	20.20 ± 0.06
SWUST b-114	-	-	48.39 ± 0.03	38.93 ± 0.06	-	-	10.13 ± 0.09	2.48 ± 0.09
SWUST b-123	-	-	45.94 ± 0.03	54.98 ± 0.03	-	-	31.22 ± 0.03	-

Data were shown by means with standard error of three replicates. “-” indicated no antagonistic activity was detected.

### 3.5. Effect of cell-free culture filtrate of SWUSTb-19 on hyphal growth, sclerotia formation, and germination of *S. rolfsii*

**Figure 5:**
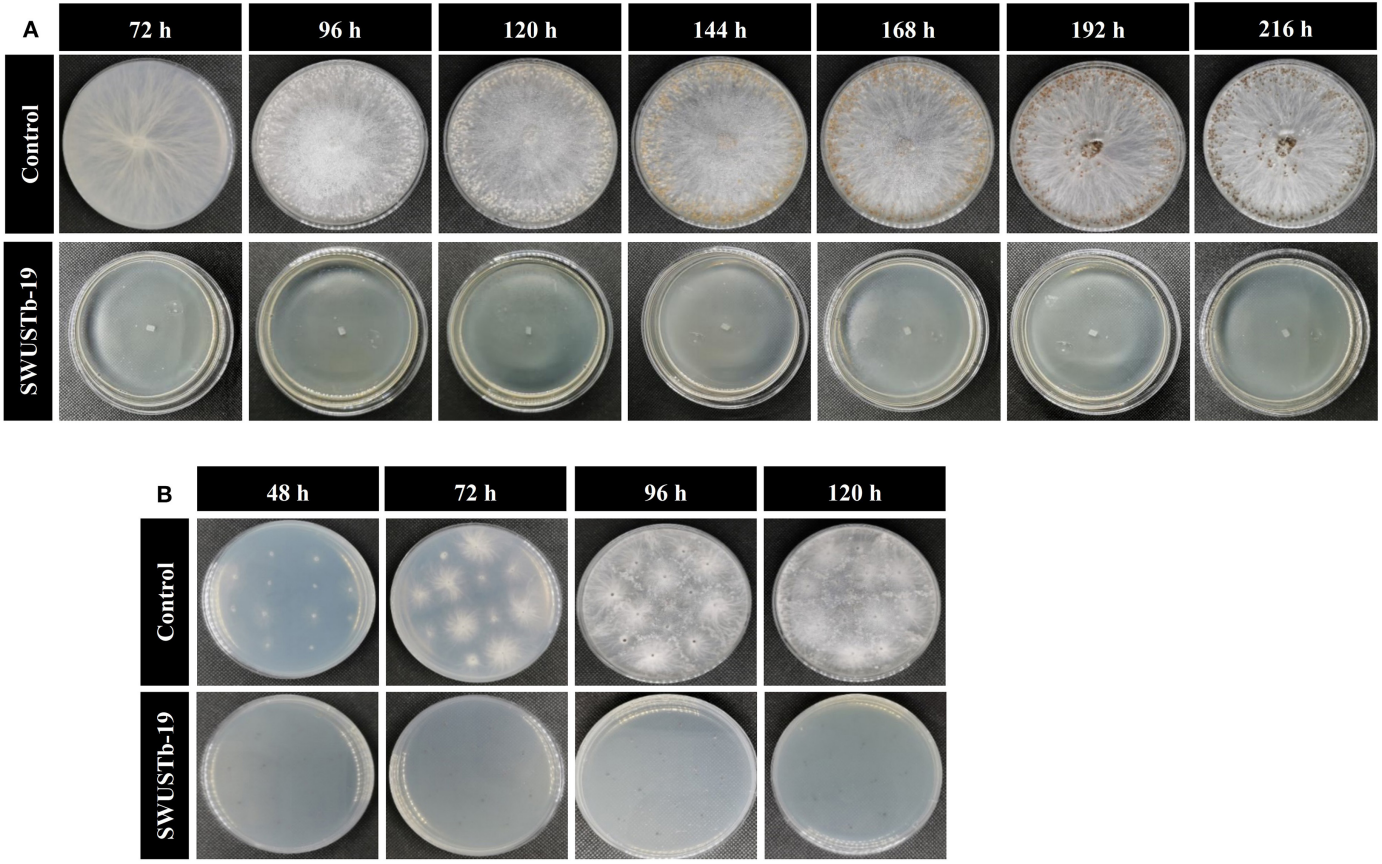
Effect of cell-free culture filtrate of SWUSTb-19 on hyphal growth, sclerotia formation, and germination of *S. rolfsii*. **(A)** Cell-free culture filtrate of SWUSTb-19 inhibited hyphal growth and sclerotia formation of *S. rolfsii* and **(B)** sclerotia germination.

To better explore the antagonistic mechanisms of SWUSTb-19, the effect of cell-free culture filtrate of SWUSTb-19 on hyphal growth, sclerotia formation, and germination of *S. rolfsii* was evaluated. As shown in Figure 5, no bacterial colony grown on the PDA plate, supplemented with cell-free culture of SWUSTb-19, indicating that the filtrating was complete. In the control treatment, *S. rolfsii* grew normally and occupied the whole plate after 72 h of incubation. The hyphae started to form sclerotia at 96 h, and a total number of 254 mature sclerotia were detected after 192 h of incubation (Figure 5A). However, cell-free culture filtrate of SWUSTb-19 completely inhibited the hyphal growth of *S. rolfsii*, and therefore, no sclerotia formed (Figure 5A).

Sclerotia of *S. rolfsii* started to germinate after 48 h incubation on the PDA plate in the control treatment. However, cell-free culture filtrate of SWUSTb-19 completely inhibited sclerotia germination even after 120 h of incubation (Figure 5B).

### 3.6. Effect of volatile compounds produced by SWUSTb-19 on the growth of *S. rolfsii*

**Figure 6:**
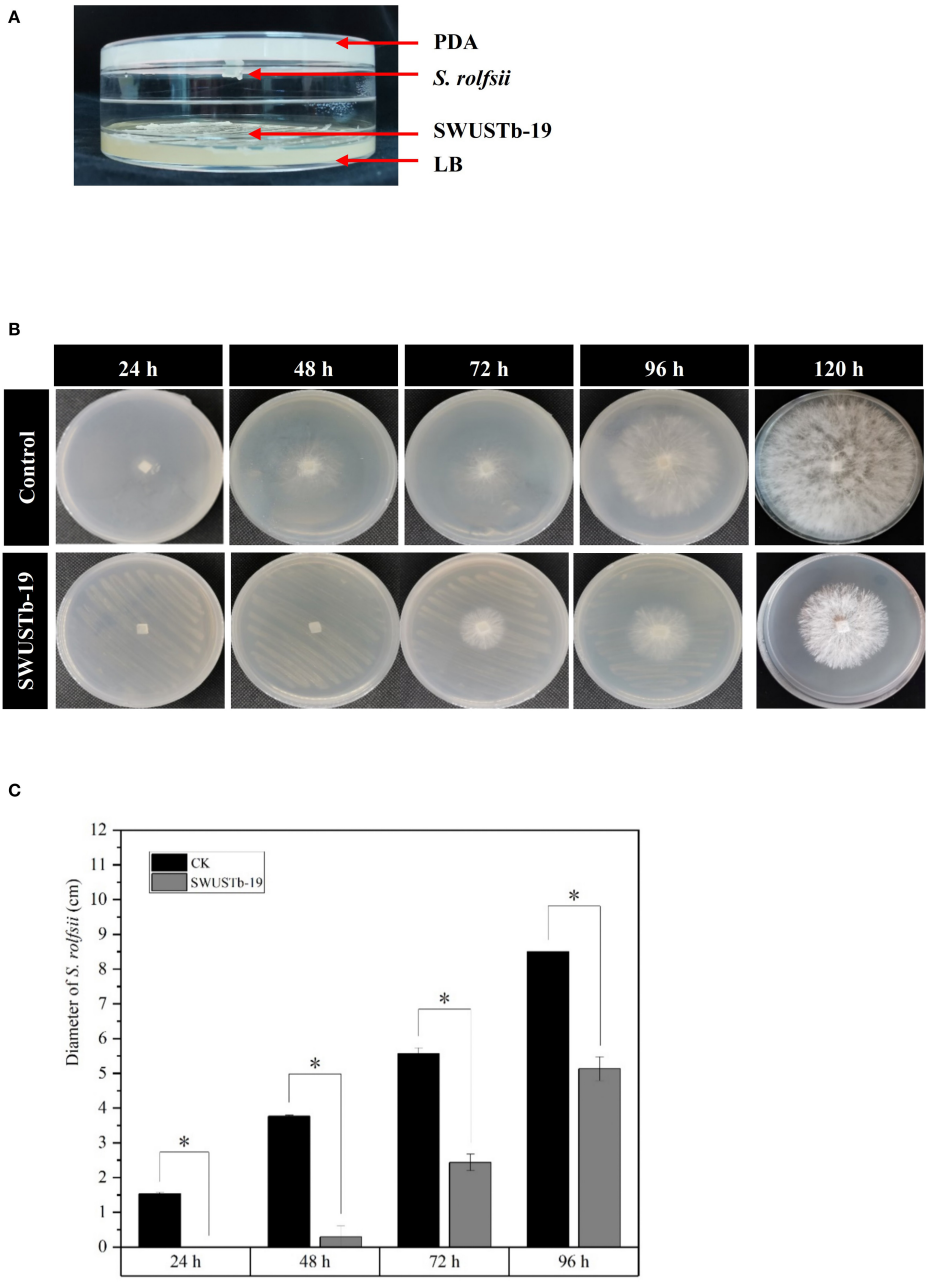
Effect of volatile compounds produced by SWUSTb-19 on the growth of *S. rolfsii*. **(A)** Schematic diagram showing two sealed base plates dual-culture assay. **(B)** Hyphal growth of *S. rolfsii* co-cultured with SWUSTb-19 in a volatile assay. **(C)** Diameter of *S. rolfsii* co-cultured with SWUSTb-19 in a volatile assay. The ^*^ symbol indicated the significant difference (*p* < 0.05) based on one-way analysis of variance with least significant difference test.

In the two sealed base plates dual culture assay, the interaction between SWUSTb-19 and *S. rolfsii* could only occur through the air in the equipment (Figure 6A). As shown in Figure 6B, *S. rolfsii* started to form new hyphae after 24 h of incubation in the control treatment, and the hyphae occupied the whole plate after 120 h of incubation. However, hyphal growth of *S. rolfsii* was postponed to 48 h post-co-culture with SWUSTb-19 (Figure 6B). Pathogenic growth was always significantly inhibited by volatile compounds produced by SWUSTb-19 over control within 96 h (Figure 6C). The inhibiting rate was 100, 92.04, 56.37, and 39.65% at 24, 48, 72, and 96 h, respectively.

### 3.7. Amplification of genes involved in antimicrobial activity

**Figure 7:**
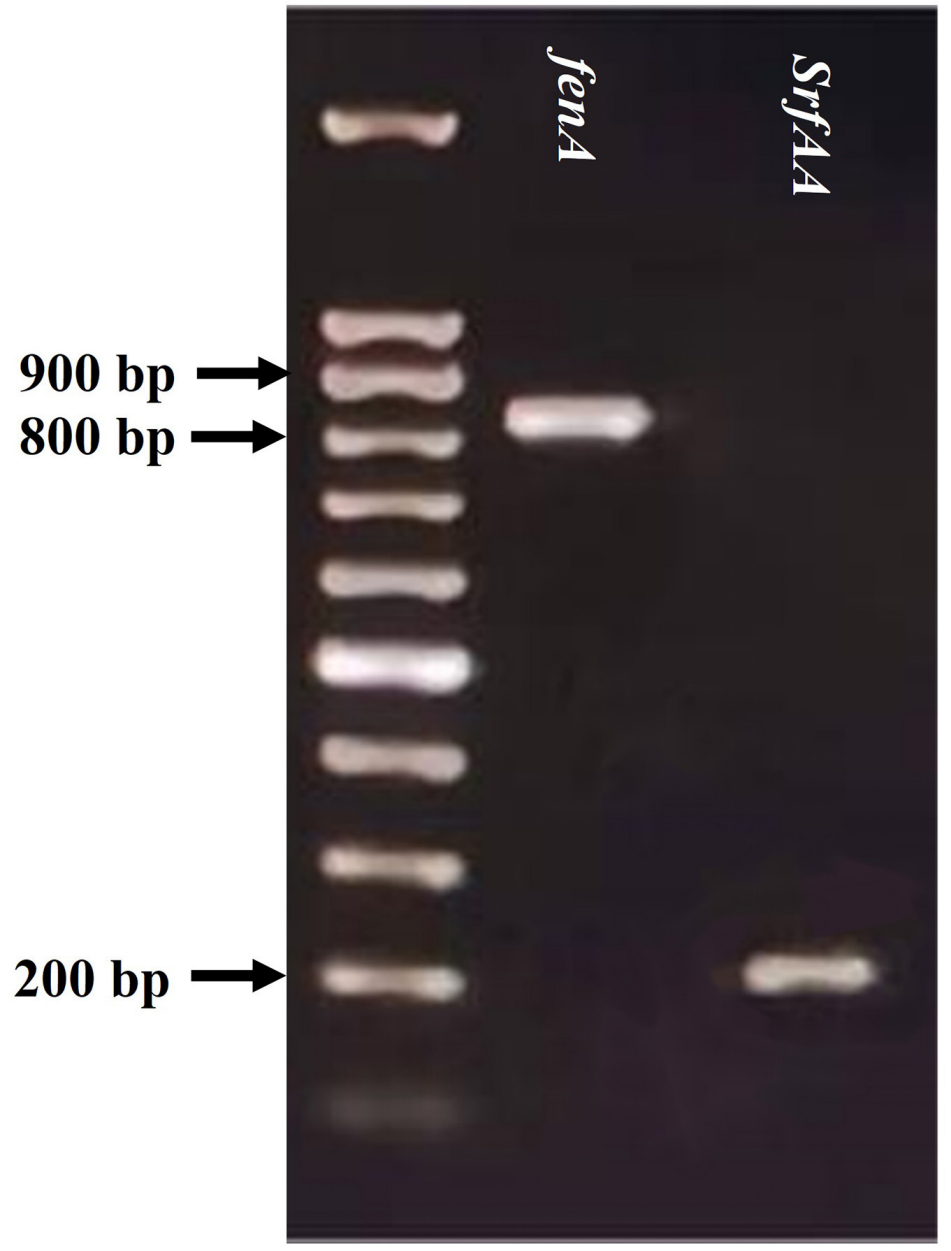
PCR detection of *fenA* and *srfAA* genes from the genomic DNA of SWUSTb-19.

The amplification of *bac* and *bacA* genes of SWUSTb-19 was failed by PCR in the current study. However, we succeed in amplifying *fenA* and *srfAA* genes, which are involved in the synthesis of fengycin and surfactin, respectively. As shown in Figure 7, PCR amplification of *fenA* and *srfAA* genes generated fragments of 850 and 200 bp, respectively, by gel electrophoresis.

### 3.8. Biocontrol and plant growth promoting potential of SWUSTb-19 by field trial

**Figure 8:**
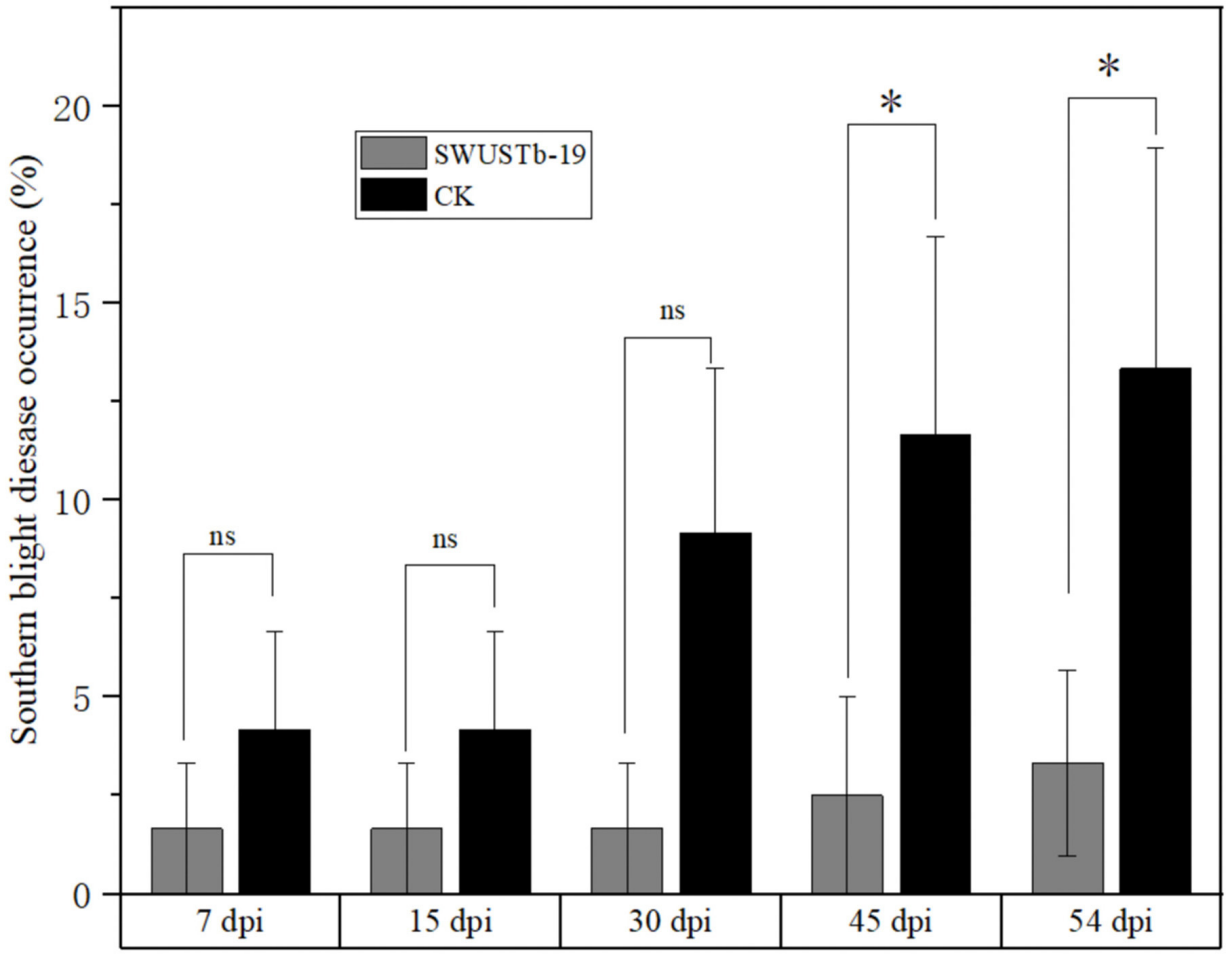
Southern blight disease occurrences of SWUSTb-19 cell fermentation culture and non-inoculation (CK) treatments by field experiments. ns indicated not significant; *indicated significant difference (*p* < 0.05) according to one-way analysis of variance with least significant difference test. dpi, days post inoculation.

Considering the great antagonistic potential of SWUSTb-19 against *S. rolfsii in vitro*, the biocontrol efficacy of SWUSTb-19 on southern blight was verified by field experiments. Fermentation culture of SWUSTb-19 was applied directly to the wounded parts of the roots after removing the redundant lateral roots to facilitate colonization of the strain. The results showed that southern blight disease occurrences by SWUSTb-19 inoculation treatment were indistinguishable from non-inoculation (CK) treatment in the first 15 days post-inoculation (dpi). However, the disease occurrence of SWUSTb-19 treatment was more than two times lower than that of the CK treatment at 30 dpi. From 45 dpi to 54 dpi, disease occurrences of SWUSTb-19 inoculation treatment were significantly reduced compared with CK (Figure 8). The control efficacy of SWUSTb-19 on southern blight was 78.5% at 45 dpi and 75.0% at 54 dpi, respectively.

After harvesting, the fresh and dry weights of stem, main root, and lateral root were recorded to evaluate plant growth-promoting capacity of SWUSTb-19. As shown in Table 4, fresh and dry weights of stem, main root, and lateral root in the SWUSTb-19 treatment were significantly higher compared with that in CK. SWUSTb-19 promoted 56.97, 43.53, and 36.40% of fresh weights of stem, main root, and lateral root, respectively, compared with CK and 52.77, 82.74, and 90.02% of dry weight of stem, main root, and lateral root of *A. carmichaelii*, respectively, compared with CK. Taken together, SWUSTb-19 showed significant biocontrol activity on southern blight and plant growth-promoting properties of *A. carmichaelii* under field condition.

**Table 4:** Effects of SWUSTb-19 cell fermentation culture on *A. carmichaelii* plant growth by field experiments.

**Treatment**	**Stem**		**Main root**		**Lateral root**	
	**Fresh weight (g)**	**Dry weight (g)**	**Fresh weight (g)**	**Dry weight (g)**	**Fresh weight (g)**	**Dry weight (g)**
SWUSTb-19	43.92 ± 2.05a	9.99 ± 0.5a	38.97 ± 2.15a	7.44 ± 0.68a	88.57 ± 8.16a	24.36 ± 2.90a
CK	27.98 ± 3.25b	6.96 ± 0.9b	28.57 ± 2.85b	4.87 ± 0.64b	48.54 ± 5.95b	12.82 ± 1.91b

Data was presented by mean ± SE. Different lower-case letters indicated significant difference (p < 0.05) according to student t-test.

## 4. Discussion

Medicinal plants harbored a tremendous number of bacterial endophytes with promising potential for plant growth promotion, antimicrobial activity, and production of bioactive compounds (Musa et al., 2020; Rat et al., 2021; Wu et al., 2021). In the current study, 124 bacterial endophytes were isolated from different tissues of *A. carmichaelii* plants collected from 10 sites in the geo-authentic area, i.e., Jiangyou, by conventional method, and their diversity and potential functions were evaluated. To our best knowledge, this is the first report characterizing culturable endophytic bacteria from *A. carmichaelii*, particularly in the geo-authentic area. Consistent with other studies (Xu et al., 2019; Sharma et al., 2020; Pang et al., 2022), *A. carmichaelii* roots captured a higher number of bacterial endophytes than other organs (Table 1). The genetic diversity of these culturable endophytic bacteria was investigated by molecular methods, i.e., 16S rDNA-RFLP analysis followed by housekeeping gene sequence analysis. 16S rDNA-RFLP can help efficiently discriminate closed species and has been widely used in the genetic diversity analysis of prokaryotes (Chen et al., 2018; Laref and Belkheir, 2022). According to 16S rDNA-RFLP analysis, 114 endophytic bacteria were clustered into 32 groups, which showed great genetic diversity. Thirty-three representative strains were selected for phylogenetic analysis using housekeeping genes sequences. Specifically, we selected two representative strains, i.e., SWUSTb-105 and SWUSTb-47 from group 3 for 16S rRNA gene sequence analysis, to verify the results of 16S rDNA-RFLP analysis. A phylogenetic tree based on full-length 16S rDNA sequence showed that SWUSTb-105 and SWUSTb-47 clustered together with 100% similarity (Figure 2), which validated the results of 16S rDNA-RFLP analysis. A total of 33 representative strains were clustered into 10 genera according to phylogenetic analysis of the 16S rRNA gene. We found that *Pseudomonas* and *Bacillus* were the dominant genera, which was consistent with other reports (Xu et al., 2019; Pang et al., 2022). In addition, other genera such as *Pantoea, Enterobacter, Klebsiella, Burkholderia, Xanthomonas, Microbacterium, Agrobacterium*, and *Sphingobacterium* were also detected. Multi-locus housekeeping gene sequence analysis (MLSA) was adopted to further explore the genetic diversity at the species level. MLSA has been proven to be an effective phylogenetic tool widely used to support and clarify the taxonomic status of bacterial species (Glaeser and Kampfer, 2015; Xu et al., 2015; Chen et al., 2018). Interestingly, we found that many strains were clustered separately by MLSA analysis including 17 representative strains which clustered into 10 clusters (Figure 3). This result indicated that *A. carmichaelii* perhaps harbored 10 potential new endophytic bacterial species. Whole genome sequencing of these strains may help confirm this hypothesis. Taken together, endophytic bacteria of *A. carmichaelii* showed great genetic diversity at the genus level, and potential new bacterial species also contribute to the composition of *A. carmichaelii* endophytic microbiome.

*A. carmichaelii* is a highly nutrient-demanding plant, and substantial amounts of fertilizers were applied to the soil for its cultivation. For example, over 150 tons per ha of stable manure were applied to the soil to prepare beds for sowing mother roots (Yu et al., 2016). In addition, at least two more times of fertilization were required during the growth periods of *A. carmichaelii*. However, the fertilizer utilization rate was quite low for *A. carmichaelii* plants; thus, most of the fertilizers resided in the soil, which facilitated continuous cropping obstacle including acidification of soil and outbreak of soil-borne diseases (Li et al., 2019b). Therefore, it is necessary to screen plant growth promoting microbial resources for environmentally friendly biofertilizers. We found that the majority of the endophytes (99 out of 124) obtained in the current study were able to produce IAA, a plant hormone that can facilitate root elongation and eventually provide a greater chance for plants to obtain nutrients (Borah and Thakur, 2020; Ben Khedher et al., 2021). A total of 50 strains were able to produce siderophore, the primary role of which is for iron uptake. Additionally, siderophore has also been proven to play a pivotal role in phytopathogen control (Gu et al., 2020). In addition, 20, two, and four strains were able to solubilize organic phosphate, inorganic phosphate, and potassium, respectively, which could promote the acquisition of phosphate and potassium by host plants. These results indicated that most of the bacterial endophytes of *A. carmichaelii* showed plant growth-promoting properties through diverse mode of actions and could be served as a new resource for biofertilizers.

Another barrier to the sustainable development of *A. carmichaelii* is soil-borne diseases (Li et al., 2019b). Screening of beneficial microorganisms working as biocontrol agents to control plant diseases has been proven to be a promising environmentally friendly alternative compared with chemical fungicides. Bacterial endophytes are among the candidates, as they are able to antagonize many pathogens. For example, seed-borne endophytic bacterial *B. velezensis* exhibited over 90% radial growth inhibition of *S. rolfsii* hyphae and significantly reduced disease incidence and severity of stem rot of peanut by pot experiments (Chen et al., 2020). *B. megaterium* OSR3 and *P. fluorescence* PF-097 showed significant antagonistic activity against *S. rolfsii* and can effectively control southern blight disease as well as increase growth and yield of Chili (Sharf et al., 2021). Endophytic *B. subtilis, B. velezensis, Leuconostoc mesenteroides*, and *Lactococcus lactis* were capable of controlling the bacterial wilt of tomato (Dowarah et al., 2021). Native endophytic *P. putida* afforded a significant reduction in common bean rust caused by *Uromyces appendiculatus* (Abo-Elyousr et al., 2021). In the current study, 20 out of 124 bacterial endophytes exhibited good antagonistic activities against *S. rolfsii* and *F. oxysporum* both *in vitro* and on root slices (Table 3). These strains also have the capacity to produce either IAA and siderophore or hydrolytic enzymes. Among the 20 strains, SWUSTb-19 showed the highest inhibition rate against *S. rolfsii* and *F. oxysporum* (Figure 4). Field experiments demonstrated that the cell fermentation culture of SWUSTb-19 applied to the roots of *A. carmichaelii*, significantly reduced southern blight occurrences compared with non-inoculation treatment at 45 and 54 dpi (Figure 8). No significant difference was detected for southern blight disease occurrences in the first 30 dpi between SWUSTb-19 inoculation and CK treatments. The possible reason was that southern blight is a root disease where pathogens first infect roots, leading to root rot followed by wilt of leaves and finally death of the plant. Therefore, when we perceived symptoms in aboveground plants, host plants have been infected severely by pathogens. It was possible that plants inoculated by SWUSTb-19 have already been infected by pathogens previously. This indicated that SWUSTb-19 should be inoculated prior to pathogen infection. However, southern blight occurrences by SWUSTb-19 inoculation treatment were kept at a stable and low level, while disease occurrences in CK increased over time (Figure 8). Li et al. (2019b, 2020) demonstrated that three strains isolated from strawberry soil, i.e., *S. pactum* Act12, *S. rochei* D74, and *P. griseofulvum* CF3 reduced significantly southern blight incidences by field experiments in the Shaanxi area, the control efficacy of which ranged from 16.5 to 31.3%. In the current study, the control efficacy by SWUSTb-19 against southern blight ranged from 60.0% (7 and 15 dpi) to 78.5% (45 dpi), which showed better biocontrol efficacy. According to phylogenetic analysis, SWUSTb-19 was classified into *Pseudomonas* genus. *Pseudomonas* was one of the dominant plant endophytic bacteria and widely used as biofertilizers and biocontrol agents (Weller, 2007; Dimkić et al., 2022). Therefore, *Pseudomonas* sp. SWUSTb-19 represents a promising biocontrol agent for the control of southern blight in *A. carmichaelii*.

**Figure 9:**
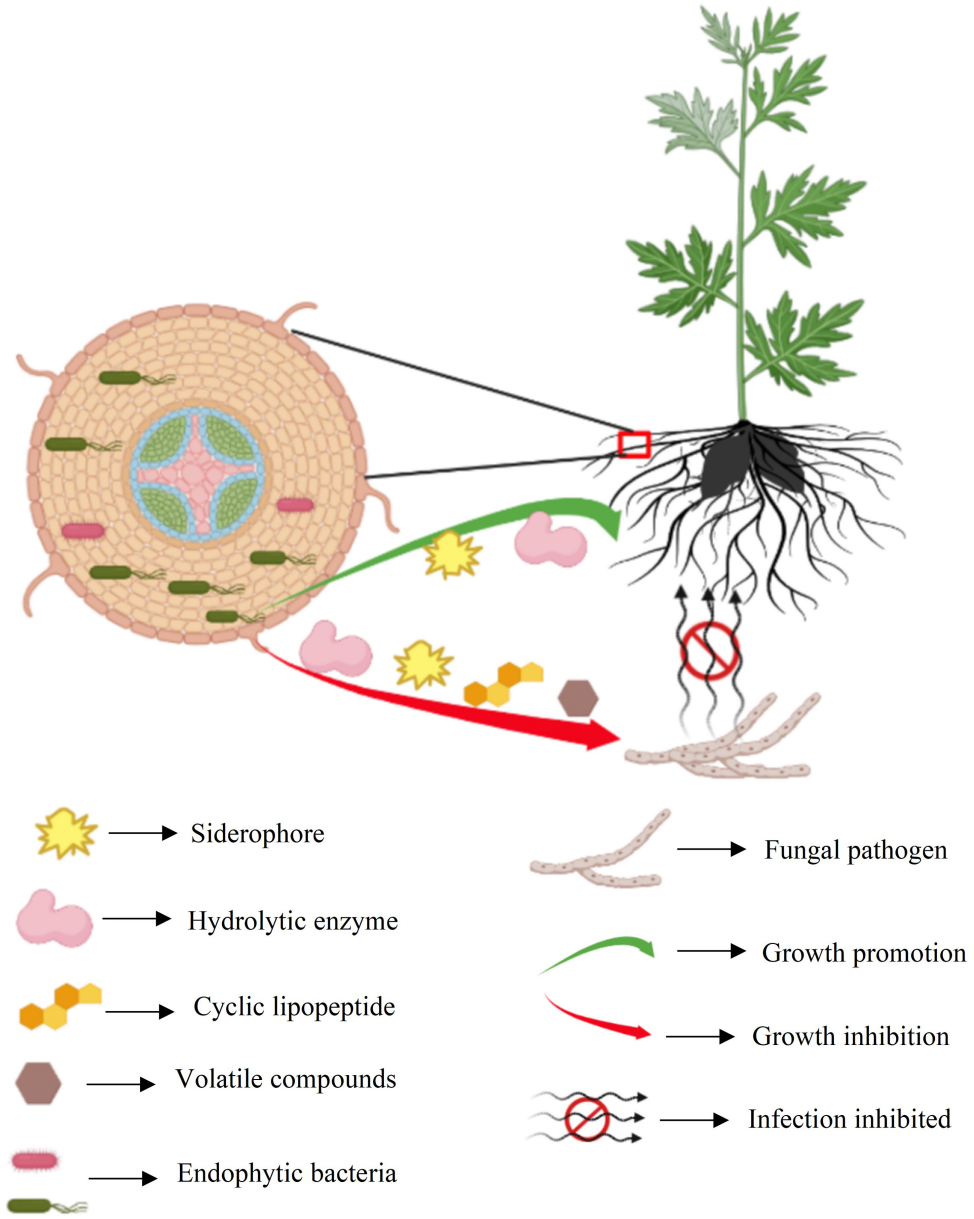
Summary schematic of endophytic bacteria SWUSTb-19 for the control of southern blight and plant growth promotion on *A. carmichaelii*.

Cell-free culture filtrate of *Pseudomonas* sp. SWUSTb-19 significantly inhibited hyphal growth, sclerotia formation, and germination of *S. rolfsii* (Figure 5). These results indicated that *Pseudomonas* sp. SWUSTb-19 secreted antifungal secondary metabolites. Hydrolytic enzymes, siderophores, cyclic lipopeptides, biosurfactins, and volatile organic compounds are among the main secondary metabolites underlying biocontrol mechanisms in *Pseudomonas* strains (Dimkić et al., 2022). We demonstrated that *Pseudomonas* sp. SWUSTb-19 was able to produce glucanase, which was able to hydrolyze glucan, one of the main components of the cell wall in pathogenic fungi (Jadhav et al., 2017). *Pseudomonas* sp. SWUSTb-19 also showed positive activity for siderophore production in CAS agar medium. Iron is an essential nutrient element that works as a cofactor for enzymes such as metalloprotease and regulatory proteins involved in many cellular processes in all organisms (Di Francesco and Baraldi, 2021). However, the amount of iron in the soil is not always available because of the low solubility (Li et al., 2019a). Many bacteria scavenge iron from the environment through the secretion of siderophores, with a high affinity for iron (Gu et al., 2020). Competition of iron by secreting siderophore represents a universal mechanism for beneficial bacteria to suppress phytopathogens (Gu et al., 2020). Whether *Pseudomonas* sp. SWUSTb-19 was able to compete for iron with pathogenic fungi under low iron concentration condition and needed further exploration. In addition, by PCR amplification, *fenA* and *srfAA* which are involved in the synthesis of fengycin and surfactin, respectively, were successfully amplified from the genomic DNA of *Pseudomonas* sp. SWUSTb-19. Fengycin is a cyclic lipopeptide with strong fungitoxic activity, specifically against filamentous fungi. Fengycin can interact with the lipid layer and to some extent retain the potential to alter the cell membrane structure and permeability of fungi (Ongena and Jacques, 2008). Surfactin is a powerful biosurfactant with the ability to self-assemble and form micelles of various sizes. Bacterial surfactin plays a pivotal role in swarming motility, biofilm formation, plant tissue colonization, and competition for nutrients and niches (Dimkić et al., 2022). In addition, by dual-culture method, we showed that *Pseudomonas* sp. SWUSTb-19 can produce volatile compounds that completely inhibited the hyphal growth of *S. rolfsii*. Aldehydes, ketones, alcohols, and sulfur-containing compounds such as methanethiol, dimethyl, and DMDS with antifungal activities have been detected in *Pseudomonas* spp. strains (Hernández-León et al., 2015). Characterization of the exact antifungal volatile compounds produced by *Pseudomonas* sp. SWUSTb-19 by gas chromatography would be of great help to elucidate the mode of action and mining useful biocontrol products. Taken together, the production of glucanase, siderophore, lipopeptides, and volatile compounds may be the potential mechanisms employed by *Pseudomonas* sp. SWUSTb-19 for antagonizing against *S. rolfsii* and promoting plant growth (Figure 9).

## 5. Conclusion

The present study explored genetic diversity, plant growth promotion, and antifungal activities of endophytic bacteria isolated from *A. carmichaelii*. A total of 124 bacterial strains were isolated from different organs of *A. carmichaelii*. These strains belonged to 10 genera including *Bacillus, Pseudomonas, Pantoea, Enterobacter, Klebsiella, Xanthomonas, Agrobacterium, Microbacterium, Rummeliibacillus*, and *Sphingobacterium*, among which *Bacillus* and *Pseudomonas* were the dominant genera based on 16S rRNA gene sequence. These endophytic bacteria showed plant growth-promoting properties including the production of IAA, hydrolytic enzymes, siderophore, and solubilization of phosphate and potassium. A total of 20 strains showed antagonistic activity against either *S. rolfsii* or *F. oxysporum in vitro* and on root slices of *A. carmichaelii*. Strain *Pseudomonas* sp. SWUSTb-19 showed promising biocontrol potential against southern blight of *A. carmichaelii* by field experiments. The possible mode of actions by *Pseudomonas* sp. SWUSTb-19 for the control of southern blight involved the production of hydrolytic enzymes, antifungal lipopeptides, and volatile compounds. Our findings indicated that *A. carmichaelii* captured diverse endophytic bacteria with great plant growth-promoting and antifungal potential.

## Data availability statement

The datasets presented in this study can be found in online repositories. The names of the repository/repositories and accession number(s) can be found in the article/Supplementary material.

## Author contributions

LZ: conceptualization, methodology, and writing—original draft. LZ and QW: data curation and formal analysis. LZ and JH: funding acquisition and project administration. QW, ML, SW, KY, and WD: investigation. LZ, KY, and JH: resources. JH: writing—reviewing and editing. All authors contributed to the article and approved the submitted version.

## References

[B1] Abo-Elyousr K.A. M. Abdel-Rahim I. R. Almasoudi N. M. Alghamdi S. A. (2021). Native endophytic *Pseudomonas putida* as a biocontrol agent against common bean rust caused by *Uromyces appendiculatus*. J. Fungi 7, 745. 10.3390/jof7090745 34575783 PMC8467904

[B2] Afzal I. Shinwari Z. K. Sikandar S. Shahzad S. (2019). Plant beneficial endophytic bacteria: Mechanisms, diversity, host range and genetic determinants. Microbiol. Res. 221, 36–49. 10.1016/j.micres.2019.02.001 30825940

[B3] Ben Khedher S. Mejdoub-Trabelsi B. Tounsi S. (2021). Biological potential of *Bacillus subtilis* V26 for the control of Fusarium wilt and tuber dry rot on potato caused by *Fusarium* species and the promotion of plant growth. Biol. Control 152, 104444. 10.1016/j.biocontrol.2020.104444

[B4] Borah A. Thakur D. (2020). Phylogenetic and functional characterization of culturable endophytic *Actinobacteria* associated with *Camellia* spp. for growth promotion in commercial Tea cultivars. Front. Microbiol. 11, 318. 10.3389/fmicb.2020.00318 32180767 PMC7059647

[B5] Carrion V. J. Perez-Jaramillo J. Cordovez V. Tracanna V. de Hollander M. Ruiz-Buck D.. (2019). Pathogen-induced activation of disease-suppressive functions in the endophytic root microbiome. Science 366, 606–612. 10.1126/science.aaw9285 31672892

[B6] Chan Y. T. Wang N. Feng Y. (2021). The toxicology and detoxification of *Aconitum*: Traditional and modern views. Chin. Med. 16, 61. 10.1186/s13020-021-00472-9 34315520 PMC8314510

[B7] Chen L. Wu Y. D. Chong X. Y. Xin Q. H. Wang D. X. Bian K. (2020). Seed-borne endophytic *Bacillus velezensis* LHSB1 mediate the biocontrol of peanut stem rot caused by *Sclerotium rolfsii*. J. Appl. Microbiol. 128, 803–813. 10.1111/jam.14508 31705716

[B8] Chen Y. X. Zou L. Penttinen P. Chen Q. Li Q. Q. Wang C. Q.. (2018). Faba Bean (*Vicia faba* L.) nodulating Rhizobia in Panxi, China, are diverse at species, plant growth promoting ability, and symbiosis related gene levels. Front. Microbiol. 9, 1338. 10.3389/fmicb.2018.01338 29973926 PMC6019463

[B9] Dai W. Chen J. Zhao D. Ye K. H. (2016). The influence of root pruning to the yield and the disease index of southern blight of *Aconitum carmichaelii* Debx. Mod. Chin. Med. 18, 1177–1179. 10.13313/j.issn.1673-4890.2016.9.020

[B10] Di Francesco A. Baraldi E. (2021). How siderophore production can influence the biocontrol activity of *Aureobasidium pullulans* against *Monilinia laxa* on peaches. Biol. Control 152, 104456. 10.1016/j.biocontrol.2020.104456

[B11] Dimkić I. Janakiev T. Petrović M. Degrassi G. Fira D. (2022). Plant-associated *Bacillus* and *Pseudomonas* antimicrobial activities in plant disease suppression via biological control mechanisms—A review. Physiol. Mol. Plant P. 117, 101754. 10.1016/j.pmpp.2021.101754

[B12] Dowarah B. Agarwal H. Krishnatreya D. B. Sharma P. L. Kalita N. Agarwala N. (2021). Evaluation of seed associated endophytic bacteria from tolerant chilli cv. Firingi Jolokia for their biocontrol potential against bacterial wilt disease. Microbiol. Res. 248, 126751. 10.1016/j.micres.2021.126751 33839507

[B13] Eljounaidi K. Lee S. K. Bae H. (2016). Bacterial endophytes as potential biocontrol agents of vascular wilt diseases—Review and future prospects. Biol. Control 103, 62–68. 10.1016/j.biocontrol.2016.07.013

[B14] Fu M. Zhang X. Chen B. Li M. Zhang G. Cui L. (2021). Characteristics of isolates of *Pseudomonas aeruginosa* and *Serratia marcescens* associated with post-harvest Fuzi (*Aconitum carmichaelii*) rot and their novel loop-mediated isothermal amplification detection methods. Front. Microbiol. 12, 705329. 10.3389/fmicb.2021.705329 34489893 PMC8417746

[B15] Glaeser S. P. Kampfer P. (2015). Multilocus sequence analysis (MLSA) in prokaryotic taxonomy. Syst. Appl. Microbiol. 38, 237–245. 10.1016/j.syapm.2015.03.007 25959541

[B16] Gorai P. S. Ghosh R. Mandal S. Ghosh S. Chatterjee S. Gond S. K.. (2021). *Bacillus siamensis* CNE6- a multifaceted plant growth promoting endophyte of *Cicer arietinum* L. having broad spectrum antifungal activities and host colonizing potential. Microbiol. Res. 252, 126859. 10.1016/j.micres.2021.126859 34536676

[B17] Gu S. Wei Z. Shao Z. Friman V. P. Cao K. Yang T.. (2020). Competition for iron drives phytopathogen control by natural rhizosphere microbiomes. Nat. Microbiol. 5, 1002–1010. 10.1038/s41564-020-0719-8 32393858 PMC7116525

[B18] Hernández-León R. Rojas-Solís D. Contreras-Pérez M. Orozco-Mosqueda M. d. C. Macías-Rodríguez L. I. Reyes-de la Cruz H.. (2015). Characterization of the antifungal and plant growth-promoting effects of diffusible and volatile organic compounds produced by *Pseudomonas fluorescens* strains. Biol. Control 81, 83–92. 10.1016/j.biocontrol.2014.11.011

[B19] Jadhav H. P. Shaikh S. S. Sayyed R. Z. (2017). “Role of hydrolytic enzymes of rhizoflora in biocontrol of fungal phytopathogens: An overview,” in Rhizotrophs: Plant Growth Promotion to Bioremediation, ed S. Mehnaz (Singapore: Springer), 183–203. 10.1007/978-981-10-4862-3_9

[B20] Javed S. Javaid A. Hanif U. Bahadur S. Sultana S. Shuaib M.. (2021). Effect of necrotrophic fungus and PGPR on the comparative histochemistry of *Vigna radiata* by using multiple microscopic techniques. Microsc. Res. Tech. 84, 2737–2748. 10.1002/jemt.23836 34028133

[B21] Khan I. H. Javaid A. (2022). DNA cleavage of the fungal pathogen and production of antifungal compounds are the possible mechanisms of action of biocontrol agent *Penicillium italicum* against *Macrophomina phaseolina*. Mycologia 114, 24–34. 10.1080/00275514.2021.1990627 34928190

[B22] Laref N. Belkheir K. (2022). Application of 16S rRNA virtual RFLP for the discrimination of some closely taxonomic-related *Lactobacilli* species. J. Genet. Eng. Biotechnol. 20, 167. 10.1186/s43141-022-00448-8 36525129 PMC9756238

[B23] Li C. Zhu L. Pan D. Li S. Xiao H. Zhang Z.. (2019a). Siderophore-mediated iron acquisition enhances resistance to oxidative and aromatic compound stress in *Cupriavidus necator* JMP134. Appl. Environ. Microbiol. 85, e01938–e01918. 10.1128/AEM.01938-18 30366993 PMC6293101

[B24] Li Y. Guo Q. He F. Li Y. Xue Q. Lai H. (2020). Biocontrol of root diseases and growth promotion of the tuberous plant *Aconitum carmichaelii* induced by actinomycetes are related to shifts in the rhizosphere microbiota. Microb. Ecol. 79, 134–147. 10.1007/s00248-019-01388-6 31165188

[B25] Li Y. Guo Q. Wei X. Xue Q. Lai H. (2019b). Biocontrol effects of *Penicillium griseofulvum* against monkshood (*Aconitum carmichaelii* Debx.) root diseases caused by *Sclerotium rolfsiii* and *Fusarium* spp. J. Appl. Microbiol. 127, 1532–1545. 10.1111/jam.14382 31304623

[B26] Liu H. Li J. Carvalhais L. C. Percy C. D. Prakash Verma J. Schenk P. M.. (2021). Evidence for the plant recruitment of beneficial microbes to suppress soil-borne pathogens. New Phytol. 229, 2873–2885. 10.1111/nph.17057 33131088

[B27] Miao L. L. Zhou Q. M. Peng C. Meng C. W. Wang X. Y. Xiong L. (2019). Discrimination of the geographical origin of the lateral roots of *Aconitum carmichaelii* using the fingerprint, multicomponent quantification, and chemometric methods. Molecules 24, 4124. 10.3390/molecules24224124 31739601 PMC6891363

[B28] Musa Z. Ma J. Egamberdieva D. Abdelshafy Mohamad O. A. Abaydulla G. Liu Y.. (2020). Diversity and antimicrobial potential of cultivable endophytic actinobacteria associated with the medicinal plant *Thymus roseus*. Front. Microbiol. 11, 191. 10.3389/fmicb.2020.00191 32226412 PMC7080825

[B29] Ongena M. Jacques P. (2008). *Bacillus* lipopeptides: Versatile weapons for plant disease biocontrol. Trends Microbiol. 16, 115–125. 10.1016/j.tim.2007.12.009 18289856

[B30] Pang F. Tao A. Ayra-Pardo C. Wang T. Yu Z. Huang S. (2022). Plant organ- and growth stage-diversity of endophytic bacteria with potential as biofertilisers isolated from wheat (*Triticum aestivum* L.). BMC Plant Biol. 22, 276. 10.1186/s12870-022-03615-8 35659526 PMC9169407

[B31] Rat A. Naranjo H. D. Krigas N. Grigoriadou K. Maloupa E. Alonso A. V.. (2021). Endophytic bacteria from the roots of the medicinal plant *Alkanna tinctoria* Tausch (*Boraginaceae*): Exploration of plant growth promoting properties and potential role in the production of plant secondary metabolites. Front. Microbiol. 12, 633488. 10.3389/fmicb.2021.633488 33633713 PMC7901983

[B32] Santoyo G. Moreno-Hagelsieb G. Orozco-Mosqueda Mdel C. Glick B. R. (2016). Plant growth-promoting bacterial endophytes. Microbiol. Res. 183, 92–99. 10.1016/j.micres.2015.11.008 26805622

[B33] Sharf W. Javaid A. Shoaib A. Khan I. H. (2021). Induction of resistance in chili against *Sclerotium rolfsii* by plant-growth-promoting rhizobacteria and *Anagallis arvensis*. Egypt. J. Biol. Pest Co. 31, 16. 10.1186/s41938-021-00364-y

[B34] Sharma A. Malhotra B. Kharkwal H. Kulkarni G. T. Kaushik N. (2020). Therapeutic agents from endophytes harbored in Asian medicinal plants. Phytochem. Rev. 19, 691–720. 10.1007/s11101-020-09683-8

[B35] Singh P. Singh R. K. Guo D. J. Sharma A. Singh R. N. Li D. P.. (2021). Whole genome analysis of sugarcane root-associated endophyte *Pseudomonas aeruginosa* B18-A plant growth-promoting bacterium with antagonistic potential against *Sporisorium scitamineum*. Front. Microbiol. 12, 628376. 10.3389/fmicb.2021.628376 33613496 PMC7894208

[B36] Wang R. Wang C. Zuo B. Liang X. Zhang D. Liu R.. (2021). A novel biocontrol strain *Bacillus amyloliquefaciens* FS6 for excellent control of gray mold and seedling diseases of ginseng. Plant Dis. 105, 1926–1935. 10.1094/PDIS-07-20-1593-RE 33289407

[B37] Weller D. M. (2007). *Pseudomonas* biocontrol agents of soilborne pathogens: looking back over 30 years. Phytopathology 97, 250–256. 10.1094/PHYTO-97-2-0250 18944383

[B38] Wu J. J. Guo Z. Z. Zhu Y. F. Huang Z. J. Gong X. Li Y. H.. (2018). A systematic review of pharmacokinetic studies on herbal drug Fuzi: Implications for Fuzi as personalized medicine. Phytomedicine 44, 187–203. 10.1016/j.phymed.2018.03.001 29526584

[B39] Wu W. Chen W. Liu S. Wu J. Zhu Y. Qin L.. (2021). Beneficial relationships between endophytic bacteria and medicinal plants. Front. Plant. Sci. 12, 646146. 10.3389/fpls.2021.646146 33968103 PMC8100581

[B40] Xu K. W. Zou L. Penttinen P. Wang K. Heng N. N. Zhang X. P.. (2015). Symbiotic effectiveness and phylogeny of rhizobia isolated from faba bean (*Vicia faba* L.) in Sichuan hilly areas, China. Syst. Appl. Microbiol. 38, 515–523. 10.1016/j.syapm.2015.06.009 26242694

[B41] Xu W. Wang F. Zhang M. Ou T. Wang R. Strobel G.. (2019). Diversity of cultivable endophytic bacteria in mulberry and their potential for antimicrobial and plant growth-promoting activities. Microbiol. Res. 229, 126328. 10.1016/j.micres.2019.126328 31521946

[B42] Yu M. Yang Y.-X. Shu X.-Y. Huang J. Hou D.-B. (2016). *Aconitum carmichaelii* Debeaux, cultivated as a medicinal plant in western China. Genet. Resour. Crop Evol. 63, 919–924. 10.1007/s10722-016-0398-8

[B43] Zhang C. Kong F. (2014). Isolation and identification of potassium-solubilizing bacteria from tobacco rhizospheric soil and their effect on tobacco plants. Appl. Soil Ecol. 82, 18–25. 10.1016/j.apsoil.2014.05.00236861984

[B44] Zhou G. Tang L. Zhou X. Wang T. Kou Z. Wang Z. (2015). A review on phytochemistry and pharmacological activities of the processed lateral root of *Aconitum carmichaelii* Debeaux. J. Ethnopharmacol. 160, 173–193. 10.1016/j.jep.2014.11.043 25479152

[B45] Zou L. Wang Q. Wu R. Zhang Y. Wu Q. Li M.. (2023). Biocontrol and plant growth promotion potential of endophytic *Bacillus subtilis* JY-7-2L on *Aconitum carmichaelii* Debx. Front. Microbiol. 13, 1059549. 10.3389/fmicb.2022.1059549 36704569 PMC9871935

