# Supplementary material for: Culturable bacterial endophytes of Aconitum carmichaelii Debx. were diverse in phylogeny, plant growth promotion, and antifungal potential
Source: Front Microbiol. 2023 May 17;14:1192932. doi: 10.3389/fmicb.2023.1192932 (PMC10229814; doi:10.3389/fmicb.2023.1192932)
Supplement: Supplementary file 1 [file Data_Sheet_1.docx]

Supplementary Table 1 Primers and reaction procedures for housekeeping gene amplification

| Gene | Primers | Reaction procedure |
| --- | --- | --- |
| 16S rDNA | 27F: 5'-AGAGTTTGATCCTGGCTCAG-3'; | 94℃ for 3 min, 30 cycles of 94℃ for 1 min, 56℃ for 1 min, 72℃ for 2 min, final extension for 72℃ 10 min |
|  | 1492R: 5'-TACGGCTACCTTGTTACGACTT-3' |  |
| *atpD* | atpD 01-F: 5'-RTAATYGGMGCSGTRGTNGAYGT-3'; | 98℃ for 2 min, 35 cycles of 98℃ for 30 s, 56℃ for 1 min, 72℃ for 45 s, final extension for 72℃ 10 min |
|  | atpD 02-R:5'-TCATCCGCMGGWACRTAWAYNGC-3' |  |
|  | atpD-0F:5'-CGGCATCAAGGTTATCGACCT G-3'; | 94℃ for 3 min, 35 cycles of 94℃ for 15 s, 55℃ for 30 s, 72℃ for 30 s, final extension for 72℃ 10 min |
|  | atpD-7R: 5'-ACCAGCGGATCCAGCTGACG-3' |  |
| *gyrA* | gyrA2F: 5'-ATGAGCGATCTGGCCAGAGA-3'; | 98℃ for 2 min, 35 cycles of 98℃ for 30 s, 57℃ for 1 min, 72℃ for 45 s, final extension for 72℃ 10 min |
|  | gyrA9R: 5'-CGCGCCTTGTTCACCTGATA-3' |  |
|  | gyrA-sch2F: 5'-ATGAGCGATCTKGCCARAGA-3'; | 98℃ for 2 min, 35 cycles of 98℃ for 30 s, 56℃ for 1 min, 72℃ for 45 s, final extension for 72℃ 10 min |
|  | gyrA-sch9R: 5'-TTCTCTTTGACCAGCTCGGC-3' |  |
| *rpoB* | rpoB-f: 5'-AGGTCAACTAGTTCAGTATGGAC-3'; | 94℃ for 3 min, 30 cycles of 94℃ for 30 s, 54℃ for 45 s, 72℃ for 30 min, final extension for 72℃ 10 min |
|  | rpoB-r: 5'-AAGAACCGTAACCGGCAACTT-3' |  |
|  | rpoB CM7-F: 5'-AACCAGTTCCGCGTTGGCCTG-3'; | 98℃ for 3 min, 35 cycles of 98℃ for 30 s, 56℃ for 1 min, 72℃ for 90 s, final extension for 72℃ 10 min |
|  | rpoB CM31b-R: 5'-CCTGAACAACACGCTCGGA-3' |  |

Supplementary Table 2 16S rDNA-RFLP analysis and phylogenetic status of culturable endophytic bacteria isolated from *A. carmichaelii*.

| Strain | 16S rDNA-RFLP | | | |  | Phylogenetic status | | |
| --- | --- | --- | --- | --- | --- | --- | --- | --- |
|  | *Hinf* Ⅰ | *Hea* Ⅲ | *Taq* Ⅰ | Group |  | 16S rDNA | MLSA | Definition |
| **SWUSTb-58** | e | A | Ⅰ | 1 |  | *Klebsiella* | *Klebsiella pasteurii* | *Klebsiella pasteurii* |
| SWUSTb-89 | a | A | Ⅰ | 1 |  |  |  |  |
| SWUSTb-62 | e | H | Ⅰ | 1 |  |  |  |  |
| **SWUST b-68** | g | A | Ⅰ | 2 |  | *Agrobacterium* | unknown | *Agrobacterium* sp. |
| **SWUSTb-105** | b | A | Ⅱ | 3 |  | *Pseudomonas* | *Pseudomonas muyukensis* | *Pseudomonas muyukensis* |
| **SWUSTb-47** | b | A | Ⅶ | 3 |  | *Pseudomonas* | *Pseudomonas muyukensis* | *Pseudomonas muyukensis* |
| SWUSTb-90 | b | A | Ⅱ | 3 |  |  |  |  |
| SWUSTb-93 | b | A | Ⅱ | 3 |  |  |  |  |
| SWUSTb-4 | b | A | Ⅱ | 3 |  |  |  |  |
| SWUSTb-43 | b | A | Ⅱ | 3 |  |  |  |  |
| SWUSTb-82 | b | A | Ⅱ | 3 |  |  |  |  |
| SWUSTb-80 | b | A | Ⅱ | 3 |  |  |  |  |
| SWUSTb-5 | b | A | Ⅴ | 3 |  |  |  |  |
| SWUSTb-76 | b | A | Ⅴ | 3 |  |  |  |  |
| SWUSTb-32 | b | A | Ⅰ | 3 |  |  |  |  |
| SWUSTb-86 | b | A | Ⅲ | 3 |  |  |  |  |
| **SWUSTb-6** | c | A | Ⅴ | 4 |  | *Klebsiella* | *Enterobacter asburiae* | *Enterobacter asburiae* |
| SWUSTb-55 | a | A | Ⅴ | 4 |  |  |  |  |
| **SWUSTb-24** | c | A | Ⅲ | 5 |  | *Bacillus* | unknown | *Bacillus* sp. |
| SWUSTb-91 | c | A | Ⅲ | 5 |  |  |  |  |
| SWUSTb-104 | f | A | Ⅲ | 5 |  |  |  |  |
| SWUSTb-54 | l | A | Ⅲ | 5 |  |  |  |  |
| SWUSTb-77 | f | A | Ⅲ | 5 |  |  |  |  |
| **SWUSTb-21** | f | A | Ⅳ | 6 |  | *Pseudomonas* | *Pseudomonas* sp-3 | *Pseudomonas* sp. |
| SWUSTb-117 | a | A | Ⅳ | 6 |  |  |  |  |
| **SWUSTb-92** | d | B | Ⅰ | 7 |  | *Bacillus* | unknown | *Bacillus* sp. |
| SWUSTb-114 | d | D | Ⅰ | 7 |  |  |  |  |
| SWUSTb-8 | d | B | Ⅰ | 7 |  |  |  |  |
| **SWUSTb-84** | d | D | Ⅸ | 8 |  | *Bacillus* | unknown | *Bacillus* sp. |
| **SWUSTb-30** | d | B | Ⅳ | 9 |  | *Bacillus* | *Bacillus subtilis* | *Bacillus subtilis* |
| SWUSTb-96 | d | C | Ⅳ | 9 |  |  |  |  |
| **SWUSTb-67** | d | A | Ⅰ | 10 |  | *Bacillus* | *Bacillus subtilis* | *Bacillus subtilis* |
| SWUSTb-99 | d | A | Ⅰ | 10 |  |  |  |  |
| SWUSTb-56 | d | A | Ⅰ | 10 |  |  |  |  |
| SWUSTb-34 | d | A | Ⅲ | 10 |  |  |  |  |
| SWUSTb-107 | d | A | Ⅳ | 10 |  |  |  |  |
| SWUSTb-10 | d | A | Ⅳ | 10 |  |  |  |  |
| SWUSTb-46 | d | A | Ⅳ | 10 |  |  |  |  |
| SWUSTb-65 | d | A | Ⅳ | 10 |  |  |  |  |
| SWUSTb-35 | d | A | Ⅱ | 10 |  |  |  |  |
| SWUSTb-64 | d | A | Ⅱ | 10 |  |  |  |  |
| **SWUSTb-75** | d | E | Ⅳ | 11 |  | *Bacillus* | unknown | *Bacillus* sp. |
| SWUSTb-53 | d | E | Ⅰ | 11 |  |  |  |  |
| **SWUSTb-17** | f | B | Ⅰ | 12 |  | *Pseudomonas* | *Pantoea deleyi* | *Pantoea deleyi* |
| SWUSTb-94 | a | B | Ⅰ | 12 |  |  |  |  |
| SWUSTb-97 | a | B | Ⅰ | 12 |  |  |  |  |
| **SWUSTb-20** | b | B | Ⅲ | 13 |  | *Rummeliibacillus* | unknown | *Rummeliibacillus* sp. |
| SWUSTb-112 | f | C | Ⅲ | 13 |  |  |  |  |
| SWUSTb-121 | f | C | Ⅲ | 13 |  |  |  |  |
| SWUSTb-61 | f | B | Ⅲ | 13 |  |  |  |  |
| SWUSTb-70 | f | B | Ⅲ | 13 |  |  |  |  |
| SWUSTb-83 | f | B | Ⅲ | 13 |  |  |  |  |
| SWUSTb-120 | f | B | Ⅳ | 13 |  |  |  |  |
| SWUSTb-50 | f | B | Ⅳ | 13 |  |  |  |  |
| SWUSTb-74 | f | B | Ⅳ | 13 |  |  |  |  |
| SWUSTb-51 | f | B | Ⅳ | 13 |  |  |  |  |
| SWUSTb-52 | f | B | Ⅳ | 13 |  |  |  |  |
| **SWUSTb-111** | c | C | Ⅲ | 14 |  | *Pantoea* | *Serratia liquefaciens* | *Serratia liquefaciens* |
| SWUSTb-116 | c | C | Ⅲ | 14 |  |  |  |  |
| SWUSTb-118 | c | B | Ⅲ | 14 |  |  |  |  |
| SWUSTb-31 | c | C | Ⅲ | 14 |  |  |  |  |
| **SWUSTb-122** | h | C | Ⅲ | 15 |  | *Pseudomonas* | *Pseudomonas* sp-2 | *Pseudomonas* sp. |
| SWUSTb-119 | h | C | Ⅲ | 15 |  |  |  |  |
| **SWUSTb-1** | g | B | Ⅵ | 16 |  | *Pantoea* | *Pantoea deleyi* | *Pantoea deleyi* |
| SWUSTb-22 | i | B | Ⅸ | 16 |  |  |  |  |
| **SWUSTb-87** | g | B | Ⅳ | 17 |  | *Pseudomonas* | unknown | *Pseudomonas* sp. |
| SWUSTb-11 | g | A | Ⅳ | 17 |  |  |  |  |
| SWUSTb-71 | g | H | Ⅳ | 17 |  |  |  |  |
| SWUSTb-13 | g | B | Ⅳ | 17 |  |  |  |  |
| SWUSTb-36 | g | B | Ⅳ | 17 |  |  |  |  |
| SWUSTb-57 | g | B | Ⅳ | 17 |  |  |  |  |
| SWUSTb-73 | g | C | Ⅳ | 17 |  |  |  |  |
| **SWUSTb-26** | b | B | Ⅴ | 18 |  | *Microbacterium* | unknown | *Microbacterium* sp. |
| SWUSTb-98 | e | C | Ⅴ | 18 |  |  |  |  |
| **SWUSTb-106** | b | C | Ⅱ | 19 |  | *Bacillus* | *Bacillus subtilis* | *Bacillus subtilis* |
| SWUSTb-27 | b | C | Ⅱ | 19 |  |  |  |  |
| SWUSTb-29 | b | C | Ⅱ | 19 |  |  |  |  |
| SWUSTb-39 | b | C | Ⅱ | 19 |  |  |  |  |
| SWUSTb-59 | b | C | Ⅱ | 19 |  |  |  |  |
| SWUSTb-48 | b | B | Ⅱ | 19 |  |  |  |  |
| SWUSTb-33 | b | D | Ⅱ | 19 |  |  |  |  |
| SWUSTb-18 | b | C | Ⅳ | 19 |  |  |  |  |
| **SWUSTb-123** | b | E | Ⅸ | 20 |  | *Bacillus* | *Pseudomonas* sp-1 | *Pseudomonas* sp. |
| SWUSTb-23 | b | D | Ⅸ | 20 |  |  |  |  |
| **SWUSTb-37** | c | D | Ⅵ | 21 |  | *Bacillus* | unknown | *Bacillus* sp. |
| SWUSTb-100 | c | D | Ⅵ | 21 |  |  |  |  |
| SWUSTb-40 | c | D | Ⅵ | 21 |  |  |  |  |
| SWUSTb-3 | a | E | Ⅵ | 21 |  |  |  |  |
| SWUSTb-16 | c | E | Ⅵ | 21 |  |  |  |  |
| **SWUSTb-79** | m | D | Ⅵ | 22 |  | *Bacillus* | unknown | *Bacillus* sp. |
| **SWUSTb-103** | a | D | Ⅶ | 23 |  | *Pantoea* | *Pantoea vagans* | *Pantoea vagans* |
| SWUSTb-44 | a | E | Ⅶ | 23 |  |  |  |  |
| **SWUSTb-38** | a | D | Ⅳ | 24 |  | *Sphingobacterium* | unknown | *Sphingobacterium* sp. |
| SWUSTb-2 | a | D | Ⅰ | 24 |  |  |  |  |
| SWUSTb-113 | a | D | Ⅲ | 24 |  |  |  |  |
| SWUSTb-15 | a | D | Ⅳ | 24 |  |  |  |  |
| SWUSTb-7 | a | D | Ⅴ | 24 |  |  |  |  |
| SWUSTb-42 | a | D | Ⅷ | 24 |  |  |  |  |
| **SWUSTb-9** | c | G | Ⅰ | 25 |  | *Enterobacter* | *Enterobacter asburiae* | *Enterobacter asburiae* |
| **SWUSTb-49** | k | E | Ⅴ | 26 |  | *Microbacterium* | unknown | *Microbacterium* sp. |
| SWUSTb-102 | c | E | Ⅴ | 26 |  |  |  |  |
| SWUSTb-81 | m | E | Ⅱ | 26 |  |  |  |  |
| SWUSTb-85 | m | E | Ⅱ | 26 |  |  |  |  |
| **SWUSTb-60** | c | E | Ⅳ | 27 |  | *Pseudomonas* | *Pseudomonas* sp-2 | *Pseudomonas* sp. |
| SWUSTb-109 | c | E | Ⅳ | 27 |  |  |  |  |
| SWUSTb-14 | c | E | Ⅳ | 27 |  |  |  |  |
| SWUSTb-19 | b | E | Ⅳ | 27 |  |  |  |  |
| SWUSTb-66 | m | E | Ⅳ | 27 |  |  |  |  |
| **SWUSTb-72** | f | E | Ⅳ | 28 |  | *Pseudomonas* | *Pseudomonas* sp-2 | *Pseudomonas* sp. |
| SWUSTb-63 | f | E | Ⅸ | 28 |  |  |  |  |
| **SWUSTb-115** | c | F | Ⅷ | 29 |  | *Pseudomonas* | *Pseudomonas asiatica* | *Pseudomonas asiatica* |
| **SWUSTb-28** | j | G | Ⅵ | 30 |  | *Pantoea* | *Pantoea vagans* | *Pantoea vagans* |
| **SWUSTb-41** | f | G | Ⅳ | 31 |  | *Xanthomonas* | unknown | *Xanthomonas* sp. |
| **SWUSTb-69** | m | H | Ⅳ | 32 |  | *Pseudomonas* | unknown | *Pseudomonas* sp. |

Strains in bold was the representative strains in the RFLP group.
